# The impact of m6A modified cargo conveyed by exosomes on the tumor microenvironment

**DOI:** 10.1007/s12672-026-05060-7

**Published:** 2026-05-21

**Authors:** Wang Yi, Qingfeng Shi, Jiahui Wu, Boxuan Zhou, Yingliang Li

**Affiliations:** 1 Department of Breast Disease Center, The First Affiliated Hospital of Nanchang University, The First Clinical Medical College of Nanchang University, Jiangxi Medical College, Nanchang, 330006 P.R. China; 2 Department of Plastic Surgery, The Second Clinical Medical College of Nanchang University, The Second Affiliated Hospital of Nanchang University, Jiangxi Medical College, Nanchang, 330006 P.R. China

**Keywords:** m6A, Exosome, Tumor microenvironment, Cancer, RNA, Protein, Biomarker, Targeted therapy

## Abstract

The dynamic evolution of tumor microenvironment (TME) is the core of cancer progression. N6-methyladenosine (m6A) modification and exosome transport are key layers of post-transcriptional gene regulation and intercellular communication, respectively. However, the intersection of these two systems, namely the transportation of m6A modified bioactive cargoes through exosomes and reshaping of TME, still lacks systematic sorting. This review aims to fill this gap. Through systematic searches of PubMed, Scopus, and Web of Science, we identified 21 original preclinical studies focused on this area over the past five years. By analyzing these literature, a conceptual framework for the “m6A-exosome axis” was established for the first time, and how it mediates TME and its effects were elucidated. We found that bioactive molecules modified with m6A can be transported to TME through exosomes. During this process, these molecules can reshape existing stromal cells and immune cells, activate tumor associated cytokines, and regulate the malignant phenotype of tumor cells. This TME remodeling further affects pathological processes such as tumor angiogenesis, epithelial mesenchymal transition (EMT), immune escape, metastasis, and drug resistance. These findings indicate that the key components of this axis not only serve as potential biomarker candidates, but also represent a promising preclinical therapeutic target. Targeting this axis provides an innovative combination strategy framework for overcoming tumor growth and metastasis.

**Supplementary Information:**

The online version contains supplementary material available at 10.1007/s12672-026-05060-7.

## Introduction

The tumor microenvironment (TME) is the setting where tumor cells develop [1]. This ecosystem encompasses not only the tumor cells but also their surrounding cellular environment, which includes various cell types such as immune-suppressive cells like cancer associated fibroblasts(CAFs), tumor-associated macrophages, immune and inflammatory cells, as well as the extracellular matrix (ECM), microvascular network, and biomolecules from adjacent regions [2, 3]. Compared to the typical microenvironment, the TME is characterized by reduced oxygen levels, acidic buildup, and an unusual immune milieu. The TME interacts with tumor cells, significantly influencing tumor growth, immune escape, metastasis, and drug resistance. As a multifaceted signaling network, the TME presents numerous cellular and molecular pathways amenable to targeted intervention. Key strategies for anti-tumor therapy encompass the inhibition of tumor-related pathways within the TME, the attenuation of cells that promote tumor growth, and the enhancement of anti-tumor immunity. These approaches are implemented in conjunction with traditional treatments, including surgery, radiotherapy and chemotherapy [1, 4].

There are over 100 chemical alterations in RNA, and methylation is the primary modification found in every type of RNA [5]. Methylation of RNA accounts for more than 60% of RNA alterations, with N6-methyladenosine (m6A) emerging as the primary modification in eukaryotic organisms, essential for controlling gene expression at the post-transcriptional level. M6A is predominantly present in mRNA’s protein-coding regions, the 3’ UTR, the area surrounding the stop codon, and in long exons [6, 7]. In tumor progression, m6A modification not only broadly influences the splicing, stability, and translation of coding gene mRNAs but also profoundly regulates the biogenesis and function of various non-coding RNAs (ncRNAs), such as microRNAs (miRNAs), long non-coding RNAs (lncRNAs), and circular RNAs (circRNAs). By these means, m6A modifications affect tumor angiogenesis, ECM remodeling, epithelial-mesenchymal transition (EMT), and immune microenvironment regulation, transforming the TME and encouraging tumor formation and metastasis [8, 9].

Exosomes, approximately 30–150 nanometers in diameter, belong to a category of extracellular vesicles characterized by a bilayer lipid membrane structure [10]. In both disease and normal states, nearly all cells, including cancer cells, release exosomes that spread throughout the body through metabolic routes [10, 11]. As important mediators of intercellular communication, the lipid bilayer membrane of exosomes effectively protects the bioactive substances encapsulated inside, ensuring that their structure remains intact during delivery and successfully reaches the target cells [12]. Furthermore, distinct proteins found on exosomes’ surface, like tetraspanins and integrins, serve as “molecular markers,” identifying and attaching to receptors on particular target cells for accurate cell-to-cell transfer [13, 14]. Based on the above characteristics of exosome, they are not only carriers of endogenous biomolecules, but also a highly promising engineering drug delivery platform. Two new types of therapeutic peptides have been developed in recent research, providing ideal payloads for exosome loading. Firstly, the “mirror peptide” composed of D-amino acids has excellent protease resistance and low immunogenicity, which can precisely interfere with key protein interactions in the TME [15]. Secondly, optimized oncolytic peptides not only retain efficient membrane lysis activity and induce immunogenic cell death, but also significantly enhance their stability and persistence of action [16]. Loading peptides with stable structures and clear mechanisms into exosome is expected to construct a novel targeted delivery system: exosome can enrich towards tumor sites through their surface labeling, while the therapeutic molecules they carry can be precisely released and exert their effects in the TME, thereby synergistically overcoming microenvironmental barriers and achieving more efficient and long-lasting anti-tumor effects. This provides a new fusion strategy for tumor targeted therapy based on exosome and TME regulation.

Based on the key roles of m6A modification and exosome transport, the functional intersection of the two, namely the intercellular transmission of m6A modified bioactive molecules through exosome, has received much attention in recent years. This dynamic process constitutes an emerging core regulatory mechanism, which is defined in this article as the “m6A-exosome axis.” This axis constitutes a key bridge for bidirectional communication between tumor cells and various cells in the microenvironment, such as immune cells, fibroblasts, endothelial cells, etc. Both parties transmit m6A modified regulatory information to each other through exosomes, thereby synergistically driving the systemic remodeling of TME. This review aims to systematically elucidate the key role of the “m6A-exosome axis” in reshaping the TME. We will first outline the basic roles of m6A modification and exosomes in cancer, and then focus on the key bioactive substances regulated by m6A and transmitted through exosomes, analyzing in depth how they affect core processes such as angiogenesis, EMT, immune escape, metabolic reprogramming, and treatment resistance. Finally, we will explore the translational potential of this axis as a novel biomarker and therapeutic target.

## Materials and methods

### Methods

This review aims to comprehensively explore the impact of bioactive substances modified by m6A on the TME after being transported via exosomes. The design and reporting of the search strategy follow the Systematic Reviews and Meta-Analyses (PRISMA) guidelines for systematic reviews [17].

### Search strategy

This study conducted an electronic search in three major databases: PubMed, Scopus, and Web of Science. The search keywords included m6A modification, exosomes, extracellular vesicles, tumor, cancer and related mesh keywords, covering the past five years up to January 30, 2026. The search used a combination of controlled vocabulary and MeSH terms, appearing in titles, abstracts, and/or subject fields. The terms were appropriately combined using the Boolean operators ‘AND’. Reference lists of included studies were also screened manually to identify additional relevant articles.

### Eligibility criteria

All original clinical/preclinical studies, if written in English and focused on the impact of m6A modified bioactive substances transported by exosomes on the TME, will be included in the analysis. Review articles, book chapters, conference abstracts, news, books, editorials, letters, commentaries, and notes were excluded from the final analysis and investigation through screening. In addition, if the study does not clearly state that m6A modification regulates bioactive substances or is not transported by exosomes or affects cancer, it is also excluded.

### Selection process

All articles extracted through a systematic search were imported into EndNote 21. After removing duplicates, two researchers independently performed the primary and secondary screening, and any discrepancies were resolved by consulting with a third researcher.

### Quality/bias assessment

The risk of bias for each included study was assessed using SYRCLE’s Risk of Bias tool, which comprises 10 questions in the domains of sequence generation, baseline characteristics, allocation concealment, random housing, blinding, random outcome assessment, incomplete outcome data, selective outcome reporting, and other sources of bias [18]. Based on the information provided in each study, including whether the experiments were in vitro or in vivo, we constructed two separate tables. The first table summarizes the analysis of the in vitro sections, while the second table presents the analysis of the in vivo sections of the included studies (the detailed results are provided in the supplementary file 1).

### Result

**Fig. 1:**
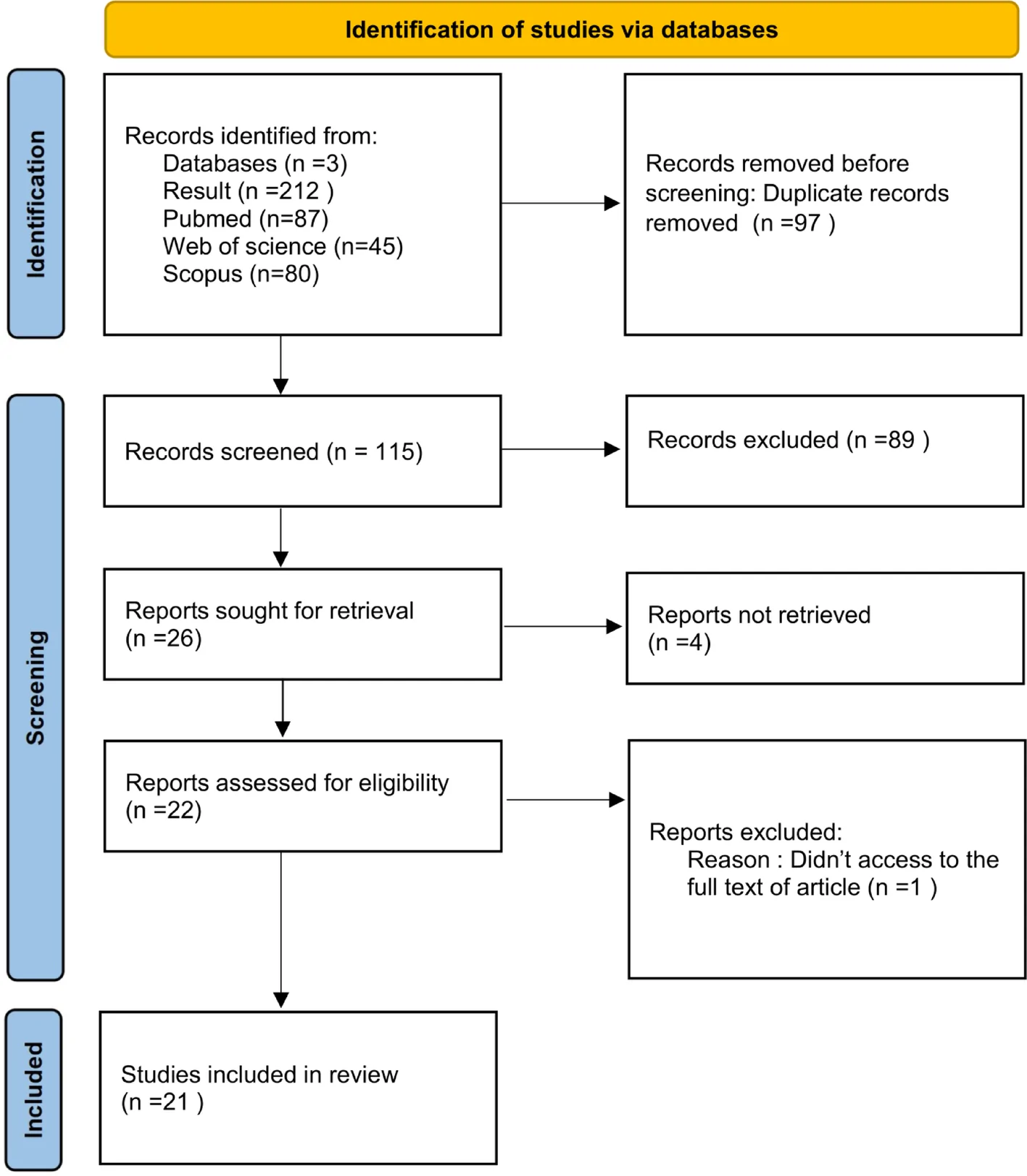
PRISMA flow diagram of the literature search

As of January 30, 2026, we have retrieved a total of 212 articles in PubMed, Scopus, and Web of Science databases. After preliminary screening, 97 duplicate articles were removed, and the remaining 115 articles entered the main screening stage through title and abstract verification. After excluding 89 articles at this stage, the final 26 articles entered full-text screening, of which 22 met the inclusion criteria, but one article could not obtain the full text due to withdrawal [19]. The latest systematic review includes a total of 21 articles. Figure 1 demonstrates the Prism diagram of the mentioned process.

## M6A modification and cancer

M6A regulators are classified into three groups: writers, erasers, and readers. The methyltransferase complex [20], known as writers, catalyzes m6A. The demethylase, referred to as an eraser, removes m6A. The RNA reader protein recognizes m6A, binds to the RNA, and carries out corresponding functions. The interaction among writers, erasers, and readers plays a role in cancer development and progression [21, 22].

The primary components of the “writers” of m6A are methyltransferase-like 3 (METTL3), methyltransferase-like 14 (METTL14), and the cofactor WTAP (Wilms’ tumor 1-associated protein) [8]. Among them, METTL3 serves as the main catalyst, while METTL14 provides the RNA binding structure. WTAP is the third essential part of this complex. Due to the absence of a conserved methylation catalytic domain, WTAP is not capable of catalyzing m6A modification. However, as an adaptor protein, it significantly influences the loading of m6A on RNA inside cells by interacting with METTL3 and METTL14 [23]. Moreover, proteins like METTL7 [24], RNA-binding motif protein 15 (RBM15), along with their homologues RBM15B [25] and Zinc finger CCCH-type containing 13 (ZC3H13) [26], have been identified as integral components of the m6A methyltransferase complex, which is essential for m6A methylation.

Methyl erasers, such as fat mass and obesity-associated gene (FTO) and AlkB homolog 5 (ALKBH5), play a role in the demethylation of m6A. FTO transforms N-methyl into hydroxymethyl groups at the m6A site [27]. ALKBH5, located near nuclear speckles and responsive to RNaseA, can eliminate methyl groups from 6-methylated adenosine, showing a preference for demethylating particular m6A-modified single-stranded RNA [28].

In addition to “writers” and “erasers,” there is another essential group in m6A known as “readers”. These can identify modifications and bind to them. Various readers can carry out distinct biological roles. Among the m6A readers, the most notable are the YTH domain family (YTHDF) 1–3 and the insulin-like growth factor 2 mRNA-binding proteins 1/2/3 (IGF2BP1/2/3) [29]. YTHDF2 is the most extensively researched m6A reader. It facilitates the degradation of m6A-dependent RNA under both normal and stress conditions [30]. YTHDF1 attaches to the m6A site near the stop codon and boosts RNA translation efficiency by interacting with eukaryotic initiation factor 3 (eIF3). In contrast to the YTHDF family, which primarily regulates Precursor mRNA splicing and translation, the IGF2BP family “readers” are tasked with recruiting RNA stabilizers to boost mRNA stability, influencing tumor growth [31]. Moreover, the “readers” of m6A modification encompass several critical proteins, including YTHDC1 and YTHDC2 [32, 33], which contain YTH domains, as well as members of the heterogeneous nuclear ribonucleoprotein family (HNRNPC, HNRNPA2B1) [34, 35], eIF3 [36], and the embryonic lethal abnormal vision-like protein 1 [37]. Each of these proteins plays a significant role in the function of m6A.

The expression and functional abnormalities of m6A regulatory factors are prevalent across various cancer types, significantly influencing tumor occurrence, progression, metastasis, and treatment response by disrupting the normal metabolism of target RNA [38]. Table 1 presents representative examples of dysregulation of m6A regulatory factors in different cancers. Overall, m6A dysregulation primarily affects tumors through two core mechanisms. One is the Pro-cancer mode, When the writer (such as METTL3) is overactive or the eraser (such as FTO) is inactivated, this may result in an abnormal increase in m6A levels in oncogene transcripts. These modified transcripts are subsequently recognized and stabilized by specific readers (such as IGF2BP2/3), leading to their overexpression. Conversely, excessive methylation of tumor suppressor gene transcription, followed by degradation by readers (such as YTHDF2), also promotes tumor development. The second is the Tumor suppression mode, in certain specific cellular environments, m6A modification can exert tumor-suppressive effects. For instance, if the function of a writer is lost or the function of an eraser is gained, resulting in decreased stability of key oncogene transcripts, this may yield anti-cancer effects.

In addition, A critical aspect of m6A regulatory function is its “context dependency.” The same regulatory factor can exhibit opposing roles across different cancers. For instance, METTL3 is frequently identified as an oncogene in hepatocellular carcinoma (HCC), lung cancer (LC), and bladder cancer (BCa) [35, 40, 41, 48, 50]. However, in certain contexts of colorectal cancer (CRC), downregulation of its homolog METTL14 demonstrates a pro-cancer effect [45]. Similarly, the demethylase ALKBH5 acts as an oncogene in osteosarcoma (OS) [44]. whereas its reduced expression correlates with poor prognosis in CRC [47]. These observations clearly suggest that the ultimate biological impact of m6A modification is not solely dictated by the regulatory factor itself, but rather hinges on whether the key target RNA it modifies functions as an oncogene or a tumor suppressor gene within a specific cellular environment.

**Table 1:** Roles of m6A in cancer

Cancer types	Dysregulated m6A	Target RNA	Reader	Change of target	Roles of m6A in cancer	References
	Regulators in cancer		Protein	RNA level		
AML	METTL14↑	MYC、MYB mRNA	ELF3	↑	Promotes tumorigenesis	[39]
HCC	METTL3↑	SOCS2 mRNA	YTHDF2	↓	Promotes tumor growth and metastasis	[40]
	METTL3/METTL14↑	CircSORE	YTHDF1/2	↑	Promotes drug resistance	[41]
BC	FTO↑	BNIP3 mRNA	YTHDF2	↓	Promotes tumor growth and metastasis	[42]
EC	WTAP↓	EGR1 mRNA	IGF2BP3	↓	Increases the aggressiveness of cancer	[43]
OS	ALKBH5↑	LncRNA PVT1	YTHDF2	↑	Promotes tumor progression	[44]
CRC	METTL14↓	LncRNA XIST	YTHDF2	↑	Promotes tumor growth and metastasis	[45]
	ALKBH5↓	Circ3823	YTHDF3	↑	Promotes tumor growth, metastasis	[46]
	ALKBH5↓	CircXPO1	IGF2BP2	↑	Promotes tumor growth, metastasis and EMT generation	[47]
NSCLC	METTL3↑	CircIGF2BP3	YTHDC1	↑	Promotes immune evasion	[48]
OC	METTL3/METTL14↑	CircNFIX	IGF2BP1-3	↑	Promotes angiogenesis and immune	[49]
					escape	
BCa	METTL3↑	MiR221/222	HNRNPA2B1	↑	Promotes tumor proliferation	[35, 50]

↑, indicates upregulation or increased level; ↓, indicates downregulation or decreased level; *AML* acute myeloid leukemia; *HCC* hepatocellular carcinoma, *BC* breast cancer, *EC* endometrial cancer, *OS* osteosarcoma, *CRC* colorectal cancer, *NSCLC* non-small cell lung cancer, *OC* ovarian cancer, *BCa* bladder cancer, *EMT* epithelial mesenchymal transition

## Exosomes and cancer

**Fig. 2:**
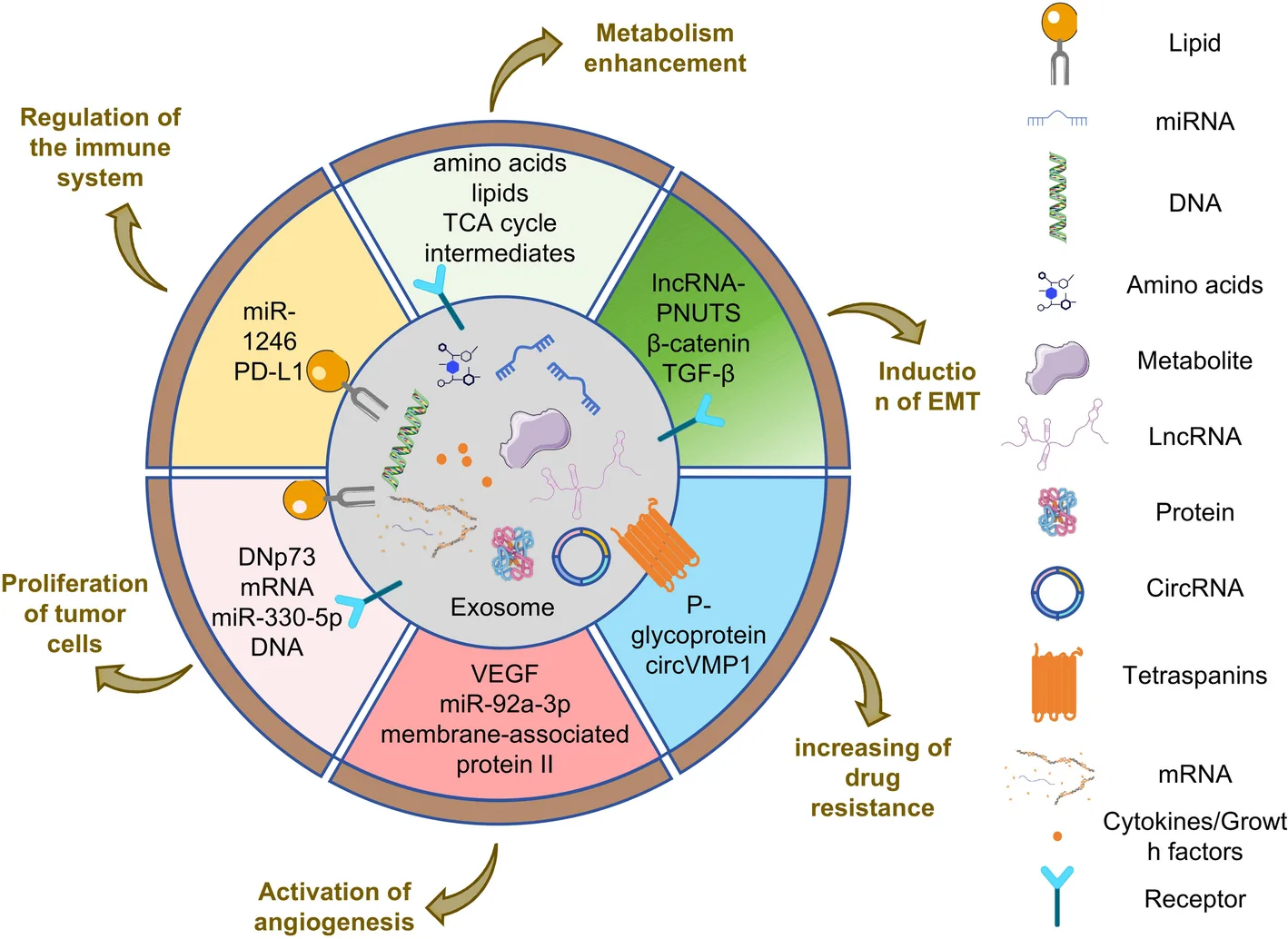
Role of exosomes in cancer. Exosome regulate tumor proliferation, activate angiogenesis, regulate the immune system, affect tumor drug resistance and cancer metabolism through autocrine/paracrine pathways, thereby promoting cancer progression and metastasis. For example, exosome can transmit growth signals and oncogenic transcripts, promoting tumor cell growth. In addition, exosomes containing pro angiogenic factors (such as VEGF, Vascular endothelial growth factor) and epithelial mesenchymal transition inducing factors (such as TGF-β, transforming growth factor-beta) can activate endothelial cells and enhance the invasive and migratory abilities of tumor cells, respectively. Furthermore, exosomes carrying immunosuppressive molecules such as Programmed Death-ligand 1 (PD-L1) and specific microRNAs (miRNAs) can inhibit the function of immune effector cells such as T cells, or induce macrophages to polarize towards M2, establishing an immunosuppressive microenvironment. In addition, exosome can also transport resistance related proteins (such as P-glycoprotein) or RNA, spreading resistance traits to sensitive cells. More importantly, exosome carrying enzymes or metabolites can also alter the metabolic pathways of recipient cells to meet the needs of tumor growth

The TME is recognized for its significant heterogeneity, comprising tumor cells, diverse stromal cells, and their surrounding microenvironment. During tumor growth and spread, tumor cells interact with the entire TME rather than acting independently [51, 52]. Increasing evidence suggests that exosomes play a crucial role in regulating the TME [53]. As key facilitators of cell-to-cell communication, exosomes influence cancer progression by transporting bioactive molecules [54, 55]. Exosomes transport various bioactive cargoes, including nucleic acids, lipids, proteins, metabolites, cytokines, and growth factors [56]. Exosome become key mediators in regulating multiple core processes of tumor progression by transporting these bioactive substances [56, 57]. As summarized in Fig. 2, these exosomal cargoes can be delivered to various receptor cells within the TME, thereby synergistically regulating tumor proliferation, angiogenesis, EMT, immune function, metabolism, and even therapeutic response. Among these goods, RNA and protein are the two most widely studied categories.

### RNA

Exosome-packaged RNA includes mRNA and ncRNA, both of which have important impacts on cancer development [58]. Current experimental studies indicate that exosome-carried mRNA holds potential for therapeutic applications, such as in gene editing, protein replacement therapy, cancer immunotherapy, and RNA-based vaccination [59, 60]. However, it is crucial to emphasize that most evidence remains preclinical, and its translation into clinical therapies is still speculative and requires further validation. Recent research indicates that the mRNA carried by exosomes also has a significant impact on the aggressiveness of cancer, as it influences cancer growth by regulating the metabolism and proliferation of cancer cells and resisting various anti-cancer therapies [61, 62]. NcRNA, another component of exosomal RNA, has emerged as a key focus in cancer research, with growing evidence of its involvement in various cancer-related processes [63]. The main roles include: the ncRNAs carried by exosomes affect the proliferation of cancer cells [64]; they influence cancer metastasis by regulating angiogenesis, EMT, and the formation of the pre-metastatic niche (PMN) [65–70]. For example, In LC, tumor cell-derived exosomes can deliver miR-5703 to tumor associated endothelial cells, promoting angiogenesis and EMT by targeting inhibitor of growth family member 4 [69]. M2 macrophage derived exosomes can drive angiogenesis through the HIF-1AN/HIF-1α/VEGFA axis [70]; they regulate immune responses by controlling immune cell function and activity, as well as the expression of immune checkpoint molecules [71–74].It is worth noting that T cells and macrophages are key components in the tumor immune microenvironment, and their functional status is directly regulated by exosome derived from the tumor. For example, MDA-MB-231 cell-derived exosome like nanovesicles can significantly affect the cytokine expression profile of CD4+ T cells, promoting their differentiation into pro-inflammatory phenotypes, suggesting that tumor vesicles have a direct role in T cell immune regulation [75]. Meanwhile, vesicles derived from M2 macrophages in the lungs can activate the second group of innate lymphocytes by delivering lncRNA 4930474H06Rik, exacerbating allergic airway inflammation [76]. These two studies revealed the vesicle mediated immune regulatory mechanisms from the perspectives of T cells and macrophages, respectively; and they enhance tumor drug resistance [77, 78]. Although significant progress has been made in the mechanism of action of ncRNAs derived from exosome in tumor development, their cross regulatory network with other key regulatory pathways, especially RNA m6A modification, is still in the preliminary exploration stage, and their synergistic or antagonistic mechanisms have not been systematically elucidated.

### Protein

Exosomes are capable of transporting a diverse array of proteins, which play pivotal roles in tumor development despite their varied effects on cancer [79]. Among these proteins, exosomes can carry carbonic anhydrase 9, membrane-associated protein II, and WNT4, all of which are known to activate endothelial cells and facilitate tumor angiogenesis [80–82]. Angiogenesis is a complex process that allows tumors to form new blood vessels, which is vital for their growth and spread [83]. Additionally, exosomes transport proteins such as β-catenin and wave protein, which are involved in the EMT process, thereby enhancing the invasiveness and metastatic potential of tumor cells [84–86]. Furthermore, exosomal proteins can target stromal and immune cells, thereby reshaping the TME. For example, proteins like extracellular matrix metalloproteinase inducer and matrix metalloproteinases can activate fibroblasts, promote ECM remodeling, and release growth factors that support tumor growth and metastasis [87]. Immune regulatory proteins, such as exosomal programmed death-ligand 1 (PD-L1), can impede T cell activity and aid in avoiding immune responses [88, 89]. Importantly, exosomal proteins have been identified as key mediators of drug resistance. The direct proteomic evidence against drug resistance in CRC provides strong support for it. A proteomic analysis of exosomes derived from CRC cells resistant to butyrate (BR) revealed unique enrichment of proteins related to proteasome pathways in BR exosomes [90, 91]. In addition, other studies have shown that exosomes can convey drug resistance-related proteins, including P-glycoprotein, multidrug resistance protein 1, lung resistance protein, and multidrug resistance-associated protein, thereby enhancing the chemoresistance of tumor cells [92–95]. The functional diversity of exosome proteins highlights their pleiotropy in regulating TME. However, many studies have focused on individual protein candidate molecules rather than integrating them into broader signaling networks. In addition, the relative contribution of exosome proteins to RNA in driving specific phenotypes has not been fully explored.

### Others

At present, multiple research teams have identified the presence of double-stranded DNA fragments and DNA mutations within exosomes [96]. Utilizing immunogold labeling techniques in conjunction with transmission electron microscopy, it has been demonstrated that certain exosomes retain DNA initially located in the cytoplasm prior to secretion [97]. Studies suggest that exosomal DNA is implicated in regulating cell survival and homeostasis, particularly in tumor cells. Blocking exosome release causes cytoplasmic accumulation of nuclear DNA, triggering DNA damage responses reliant on reactive oxygen species. This can result in cell cycle arrest or apoptosis [97]. Major angiogenesis-stimulating factors, including interleukin-8 (IL-8), platelet-derived growth factor, fibroblast growth factor (FGF), tumor necrosis factor-alpha, bFGF, and vascular endothelial growth factor (VEGF), are transported via exosomes, thereby facilitating tumor vascularization [98, 99]. Key elements of EMT, such as transforming growth factor-beta (TGF-β), IL-6, and hypoxia-inducible factor 1-α, can also be conveyed by exosomes, inducing tumor epithelial cells to undergo EMT, which subsequently leads to distal metastasis of the tumor [85, 86]. Furthermore, the role of exosomal metabolites in cancer is increasingly being recognized. Cancer-associated fibroblast-derived exosomes transport metabolic cargos, including amino acids, lipids, and tricarboxylic acid cycle intermediates. Upon uptake by prostate and pancreatic cancer cells, these exosomes enhance glycolysis and glutamine-dependent reductive carboxylation, thereby promoting tumor growth under conditions of nutrient deprivation or stress [100, 101].

Exosomes play a critical role in the initiation and progression of cancer, highlighting their significant therapeutic potential. However, current research lacks a comprehensive understanding of the specific functions of certain components (such as metabolites and DNA), and their complex biological effects and clinical applications require further investigation.

## The impact of m6A modified cargo conveyed by exosomes on the TME

Exosomes, as key carriers of intercellular communication, can transport various bioactive substances modified by m6A, including miRNAs, lncRNAs, circRNAs, and proteins. The m6A modification profoundly influences the functions of these molecules after exosomal delivery by regulating their stability, processing maturity, translation efficiency, and sorting processes, thereby systematically reshaping the TME and driving malignant processes such as immune evasion, angiogenesis, metastasis, and drug resistance. To clearly present the common patterns of this complex network, the core regulatory modes and functional impacts of the four categories of substances are summarized in Table 2, followed by detailed elaboration and comparative analysis.

### MiRNA

MiRNAs are small, ncRNA strands, 18–24 nucleotides in length, that control gene expression post-transcriptionally by creating RNA-induced silencing complexes (RISC) [102]. The m6A modification controls the biosynthesis and maturation of miRNAs [35, 103]. Initially, the DNA is transcribed into primary miRNAs (pri-miRNAs) in the nucleus, and then the microprocessor complex consisting of DiGeorge Cirtical Region 8 (DGCR8) and DROSHA processes pri-miRNAs to precursor miRNAs (pre-miRNAs). These pre-miRNAs are finally broken down into mature single-stranded miRNAs by the Dicer enzyme in the cytoplasm [104]. In this process, the transcriptional regulators METTL3 and hnRNPA2B1 play key roles. METTL3 modifies the pri-miRNAs by adding an m6A mark, and hnRNPA2B1 interacts with the m6A in pri-miRNAs, eventually recruiting DGCR8. In this way, DGCR8 is able to identify its substrates and bind to them, resulting in the enhanced production of miRNAs [35, 103]. In addition, studies have shown that hnRNPA2B1 can also regulate miRNA sorting in exosome [105].

Through this regulatory axis, METTL3-mediated m6A modification not only controls the maturation of specific miRNAs but also influences their subsequent loading into exosomes. According to the included preclinical studies, Once these exosomal miRNAs delivered to recipient cells in the TME, they execute distinct yet converging programs that collectively foster a pro-tumor niche. The most recurrent outcome is the induction of an immunosuppressive macrophage phenotype (M2 polarization). In cell model of gastric cancer (GC), METTL3-dependent maturation of the miR-17-92 cluster enables its exosomal transfer to peritoneal macrophages. There, it activates SRC signaling, leading to the production of immunosuppressive cytokines, inhibition of T-cell activity, and the establishment of a PMN. This suggests that it may promote peritoneal dissemination [107]. Similarly, In cell model of lung adenocarcinoma (LUAD), hnRNPA2B1-mediated m6A modification promotes the biogenesis and exosomal packaging of miR-3153, which, upon uptake by macrophages, drives M2 polarization via the JNK pathway, further contributing to an immunosuppressive TME [109].

**Table 2:** The impact of exosomal transport of m6A modification-related bioactive substances on the TME

Category of bioactive substance	Main m6A regulators	Typical regulatory mechanism	Primary target cell types in TME	Overall biological effect on TME	Cancer types	References
miRNA	METTL3,	Promotes pri-miRNA	Lymphatic/ Vascular	Promotes lymphangiogenesis	ESCC	[106]
	hnRNPA2B1	processing and	Endothelial Cells,	/angiogenesis; induces M2	GC, LC	[107, 108]
		maturation; Regulates	Macrophages, T cells	macrophage polarization;	LUAD	[109]
		miRNA functional		inhibits T cell function		
		activity				
lncRNA	METTL3/7/14,	Regulating the stability	Tumor cells,	Regulates tumor cell proliferation	HCC	[110, 111]
	ZC3H13, FTO,	degradation, and	Macrophages	and angiogenesis; induces M2	GBC	[112]
	IGF2BP2/3	sorting of lncRNAs in		Macrophage polarization; mediates	MM, BC	[113, 114]
		exosome		chemotherapy resistance	O/LSCC	[115, 116]
					PCa	[117]
circRNA	METTL3/14,	promotes the	CD8 + T cells,	Induces T cell exhaustion; pro-	HCC	[118, 119]
	WTAP,	translation and stability	Macrophages,	motes M2 macrophage polariza-	NSCLC	[120]
	IGF2BP3,	of circRNA	Tumor cells	tion; facilitates EMT; activates	BCa	[121]
	YTHDC1			pro-oncogenic signaling pathways		
Protein	METTL3, FTO,	Enhances stability and	CD8 + T cells,	Inhibits T cell activity; induces M2	HCC	[122]
	ALKBH5,	translation efficiency	Macrophages,	macrophage polarization;	CCa	[123]
	IGF2BP2	of target mRNA,	Tumor/Stromal cells	inactivates tumor suppressor	CRC	[124]
		leading to upregulated		proteins; directly regulates	ESCC	[125]
		protein expression		transcription	LUAD	[126]

*ESCC* esophageal squamous cell carcinoma, *GC* gastric cancer, *LC* lung cancer, *LUAD* lung adenocarcinoma, *HCC* hepatocellular carcinoma, *GBC* gallbladder cancer, *MM* multiple myeloma, *BC*breast cancer, *OSCC* oral squamous cell carcinoma, *LSCC* laryngeal squamous cell carcinoma, *PCa* prostate cancer, *NSCLC* non-small cell lung cancer, *BCa* bladder cancer, *CCa* Cervical cancer, *CRC* colorectal cancer, *TME* tumor microenvironment, *EMT* epithelial mesenchymal transition

A distinct but related mechanism is exemplified in vitro studies of LC, where METTL3-mediated m6A modification of miR-125a-5p does not alter its exosomal abundance but impairs its loading into the functional RISC. This “qualitative” defect diminishes its ability to suppress the immune-regulatory target Immunoglobulin superfamily member 11 (IGSF11). This is positively correlated with tumor immune escape [108]. This case underscores that m6A can regulate not only miRNA “quantity” but also its functional competence-a layer of control that may be missed by expression-level analyses alone. Beyond immune cells, exosomal m6A-modified miRNAs can directly activate other stromal compartments. In esophageal squamous cell carcinoma (ESCC) cells model, METTL3 guided maturation of miR-320b and its exosomal delivery to human lymphatic endothelial cells stimulates lymphangiogenesis. This suggests that it may promote lymphatic metastasis [106].

In summary, despite the diversity of cancer types (ESCC, GC, LC, LUAD) and specific miRNA molecules involved, the collected evidence reveals a convergent paradigm: the METTL3-initiated m6A-exosome-miRNA axis predominantly remodels the immune landscape of the TME. The most consistently observed outcomes are M2 macrophage polarization, functional impairment of adaptive immunity, and the fostering of pre-metastatic niches. These processes suggest the possibility of promoting immune suppression and metastasis. In addition, While the quantitative control of miRNA abundance remains the dominant reported mechanism, the qualitative regulation of miRNA activity (as in LC) presents an important, though less validated, dimension. Future studies are needed to systematically test the generality of these patterns across broader cancer contexts and to elucidate the precise molecular cascades that link m6A-modified exosomal cargo to specific TME alterations.

### LncRNA

LncRNAs are transcripts exceeding 200 nucleotides in length [127]. They can serve as prognostic biomarkers and regulatory factors for the TME [128]. Its value may stem from different molecular sources. For example, lncRNA expression features from specific biological processes such as copper dependent cell death have been shown to robustly predict patient survival and immune therapy response in cancers such as oral squamous cell carcinoma (OSCC) [129]. In addition, the post transcriptional fate and function of many lncRNAs are also heavily influenced by epigenetic transcriptional modifications, most notably m6A. The m6A modification determines their fate after exosomal delivery by regulating their stability, degradation, and sorting [30, 31, 130]. This section will systematically review the association between m6A modified exosomal lncRNAs and TME features such as immune suppression and angiogenesis by acting on different target cells, based on existing research, and explore their potential roles in tumor progression.

Preclinical studies have shown that one of the main roles of these exosomal lncRNAs appears to be driving immune suppression in the TME, with macrophages being the primary target cell population. For instance, in breast cancer cell model, m6A-modified LINC00657 is delivered to macrophages via exosomes, inducing their polarization toward the immunosuppressive M2 phenotype, thereby weakening the antitumor immune response [114]. Similarly, in in vitro studies of HCC, m6A-modified SLC16A1-AS1 also promotes M2 polarization of macrophages through the exosome pathway [111]. These cases reveal a common strategy by which tumor cells actively “educate” macrophages via the m6A-exosome axis, transforming them into pro-tumor accomplices. Furthermore, this axis serves as a key driver of tumor angiogenesis by directly acting on endothelial cells. In cell model of gallbladder cancer, the m6A reader IGF2BP2 stabilizes the lncRNA TRPM2-AS, which is delivered to endothelial cells via exosomes. Within endothelial cells, TRPM2-AS activates the NOTCH1 signaling pathway, upregulates pro-angiogenic factors such as VEGFA, and directly drives tumor angiogenesis [112]. This mechanism is independent of immune cells, demonstrating a direct pathway through which tumors “command” stromal cells (endothelial cells) via exosomal lncRNAs to provide nutritional support for their growth.

More importantly, this regulatory axis may form a communication pathway for metabolic reprogramming and drug resistance transmission between the distal matrix and tumor cells. In in vitro experiments of multiple myeloma, METTL7A in adipocytes mediates the m6A modification of lncRNAs LOC606724 and SNHG1. These lncRNAs are exosomal-transmitted from adipocytes to myeloma cells, thereby enhancing c-Myc translation and leading to chemotherapy resistance in tumor cells [113]. This discovery reveals how the metabolic matrix (adipocytes) in the TME remotely confers drug resistance to tumor cells via the m6A-exosome axis, serving as a paradigm of TME involvement in therapeutic resistance.

It is worth noting that the regulatory effect of this axis on tumor malignant behavior exhibits significant heterogeneity, which depends on the specific regulatory factors and molecular environment in the context of its action. It is precisely this “background dependent” characteristic that reflects the complexity of its regulatory network. In vitro studies of hepatitis B virus-associated HCC, exosomes derived from M2 macrophages deliver m6A-modified lncRNA MAAS to HCC cells, stabilizing the c-Myc protein and directly driving its proliferation [110]. In vitro studies of laryngeal squamous cell carcinoma, exosomes originating from CAFs deliver m6A-modified lncRNA TUC338 to tumor cells, upregulating the expression of the oncogene CBX2 and promoting cell proliferation [116]. These two cases, together with the aforementioned pattern of lncRNA “educating” macrophages and stromal cells derived from tumor cells [111, 114], collectively form a positive feedback loop. In contrast, FTO upregulates the expression of lncRNA FLG-AS1 in OSCC cells through demethylation. According to clinical sample analysis, this lncRNA is also positively correlated with tumor progression after delivery through exosomes [115]. It is worth noting that this is in contrast to the situation of prostate cancer (PCa): in vitro studies of PCa, m6A modification mediated by the writing factor ZC3H13 upregulates the lncRNA A1BG-AS1, and exosome delivery can inhibit the proliferation of PCa cells, suggesting that this RNA may have an inhibitory effect on tumor malignant progression [117]. These interrelated and seemingly contradictory findings strongly indicate that the regulation of m6A modification on exosomal lncRNA function may have a highly context-dependent. The ultimate impact on the TME (promoting or inhibiting cancer) cannot be simply predicted by “addition or removal of modifications,” but rather depends on specific cancer types, cell sources, and downstream signaling pathways.

Although the aforementioned cases reveal the multifaceted role of the m6A-lncRNA axis in reshaping the TME, most studies have isolatedly focused on the function of individual lncRNAs in specific scenarios, lacking systematic exploration of how different lncRNAs are co-regulated and form functional networks within the same tumor model. For example, does METTL3 simultaneously regulate pro-angiogenic lncRNAs and pro-immunosuppressive lncRNAs? Is there a hierarchical relationship between them? These questions remain unanswered.

### CircRNA

CircRNA represents a distinct category of ncRNA characterized by its circular structure, which is produced through a process known as back-splicing [131]. M6A modification can drive the translation of circRNAs, and the m6A within circRNAs can operate as an internal ribosomal entry site, thus allowing a cap-independent translation [132]. METTL3 and METTL14 promote the m6A-dependent translation of circRNAs, whereas m6A demethylation by FTO negatively regulates it. YTHDF3 and eIF4G2 are major players in this ‍‌pathway.

Through systematic literature search and analysis, four preclinical studies have been identified that explicitly elucidate the specific regulatory mechanisms of m6A-modified circRNAs transported via exosomes on the TME. Although limited in number, these studies clearly reveal a positive correlation between this axis and immune suppression and tumor progression.

In terms of driving immune suppression, two studies revealed a common mechanism centered on the “m6A-circRNA-reader protein” complex. In HCC cell model, m6A-modified circCCAR1 is delivered to CD8 + T cells via exosomes, where it collaborates with the reader protein IGF2BP3 to stabilize PD-1 mRNA, directly inducing T cell exhaustion and associated with resistance to PD-1 therapy [118]. In cell and animal models of non-small cell lung cancer, exosome-delivered circPACRGL is taken up by macrophages, where it stabilizes YAP1 mRNA by binding to IGF2BP2, thereby driving macrophage M2 polarization and positively correlated with immune suppression [120]. These two studies suggest that m6A circRNAs targeting immune cells may recruit specific reading proteins as molecular scaffolds to stabilize key immune checkpoint or regulatory factor mRNA at the post transcriptional level, thereby continuously inhibiting anti-tumor immune responses.

In terms of directly promoting tumor progression, the other two studies demonstrated distinct pathways of action, highlighting the diversity of mechanisms. In the study of BCa in vitro, m6A-modified circSLC38A1 is transmitted between tumor cells via exosomes, whose function is to activate TGF-β2 transcription, thereby inducing EMT. Animal models further suggest that it may promote tumor invasion and metastasis [121]. In the cell model of HCC, exosome-delivered m6A-modified circ-CDYL has been shown to simultaneously activate multiple oncogenic signaling pathways, including HDGF-PI3K-AKT-mTOR and HIF1AN-NOTCH2. This suggests that it may drive the development of liver cancer [119]. Unlike the former, which primarily regulates a single pathway (TGF-β), circ-CDYL functions more akin to a “signal hub,” influencing multiple core pathways simultaneously. These two studies indicate that targeting m6A-circRNAs within tumor cells may directly contributes to malignant phenotypes, but the specific downstream nodes and signaling networks may vary depending on the circRNA.

Currently, research heavily relies on mechanistic exploration of individual molecules. Whether these reported circRNAs are universally representative within the complex exosomal circRNA repertoire, and whether undiscovered, functionally redundant, or antagonistic members exist, awaits future comprehensive omics analysis and functional screening.

### Proteins

Exosome-delivered ncRNAs primarily function as regulatory elements of gene expression, indirectly influencing the functions of recipient cells through post-transcriptional regulation (Table 2). In contrast, proteins are positioned downstream in functional execution, often serving as terminal effector molecules that exert direct effects. Exosome-mediated delivery of m6A-regulated proteins represents a more direct and rapid mode of TME remodeling-namely, the “intercellular transfer” of effector molecules themselves. Currently, only a limited number of proteins have been confirmed to be regulated by m6A and transported via exosomes, including Glycoprotein Non-metastatic Melanoma protein B (GPNMB), interferon gene exosome stimulator (STING), N-acetyltransferase 10 (NAT10), Krüppel-Like Factor 6 (KLF6), and Mortalin (HSPA9, a member of the HSP70 gene family). Although their numbers are limited, these proteins functionally encompass multiple aspects ranging from immunomodulation to intracellular nuclear signaling intervention, preliminarily revealing the potential and diversity of this pathway (Table 2).

Firstly, in terms of immunosuppression, exosome proteins can directly act as ligands or signaling molecules on immune cells. In in vivo and in vitro studies of HCC, FTO enhances the stability of GPNMB mRNA through demethylation, leading to upregulation of GPNMB protein expression and its sorting into exosomes. Exosome-bound GPNMB acts as a ligand, directly binding to the SDC4 receptor on the surface of CD8 + T cells, thereby inhibiting T cell activation and function [122]. This mechanism differs from circRNA indirectly inducing T cell exhaustion by stabilizing PD-1 mRNA at the post-transcriptional level [118], representing a more direct, receptor-ligand-mediated surface signal suppression. Similarly, in CRC cell model, the reader IGF2BP2 stabilizes STING mRNA via m6A-dependent mechanisms, enabling STING protein delivery to macrophages via exosomes. Upon uptake, exosomal STING protein directly activates its intrinsic signaling pathway within macrophages, inducing M2 phenotype polarization [124]. Here, exosomes transport core adaptor proteins of the signaling pathway, directly initiating specific polarization programs within recipient cells.

Secondly, exosomal proteins can mediate metabolic and epigenetic reprogramming. In ESCC cell model, the writer METTL3 upregulates the expression of N-acetyltransferase 10 (NAT10) through m6A modification. After being taken up by macrophages, exosomal NAT10 functions directly as a metabolic/epigenetic modifier enzyme, altering the stability of target mRNA (e.g., FASN) in macrophages by catalyzing RNA modifications such as ac4C, thereby remodeling lipid metabolism and driving M2 polarization [125]. This demonstrates how the m6A-exosome axis may directly alters the metabolic state of recipient cells by transferring intact enzymatic functions.

Finally, this axis can directly regulate core survival and proliferation signaling in tumor cells. In in vitro studies of cervical cancer, the writer METTL3-mediated m6A modification significantly enhances the translation efficiency of the molecular chaperone protein Mortalin (HSPA9). After being taken up by recipient cells, the exosomal Mortalin acts as a key signal-interfering protein, directly binding to and blocking the nuclear translocation of the tumor suppressor protein p53, thereby inactivating it and promoting cell cycle progression while resisting treatment-induced senescence [123]. This achieves functional transfer of key pathway proteins, directly altering the fate determination of recipient cells. Similarly, in lung adenocarcinoma cell model, ALKBH5-mediated KLF6 mRNA demethylation reduces its stability and expression. Inhibition of ALKBH5 enhances the m6A modification of KLF6, promoting its expression in M1 macrophages and sorting into exosomes. The exosome-delivered KLF6 protein, once taken up by lung cancer cells, enters the nucleus as a transcription factor, directly regulating the transcription of downstream genes, thereby inhibiting tumor cell proliferation, migration, and invasion [126]. Here, exosomes transport transcription factors that can directly regulate the expression of downstream target genes, including cyclins.

In summary, the five m6A-regulated exosomal proteins reported in current studies exhibit functional patterns distinct from those of ncRNAs: some act as ligands to directly inhibit immune cells (GPNMB), others serve as signaling molecules to initiate specific programs (STING), some function as metabolic enzymes to remodel cellular states (NAT10), some act as transcription factors to directly reprogram gene expression (KLF6), and others function as pathway proteins to interfere with core signaling (Mortalin). These findings preliminarily reveal the unique value of proteins as direct effector molecules in this axis. However, related research is still in its infancy, and the aforementioned cases represent only preliminary explorations. Future studies are needed to identify other m6A-regulated exosomal proteins and elucidate the precise mechanisms by which m6A modifications influence their post-translational secretion and specific sorting into exosomes.

## M6A-related biologically active substances in exosome as potential biomarker candidates

Liquid biopsy has recently become a noninvasive and convenient method for diagnosing cancer [133, 134]. Exosomes, in particular, offer distinct benefits in specific areas compared to other liquid biopsy sources like circulating tumor cells and circulating tumor DNA. They not only have higher stability and content in the blood circulation, but can also be obtained from almost all bodily fluids [135, 136]. Consequently, proteins and nucleic acids within exosomes have been identified as innovative diagnostic and prognostic tools for a range of cancers [137]. In addition, research has shown that m6A modified ncRNA and proteins can be transported by exosomes, and their levels vary in cancer patients compared to healthy individuals, making them promising candidate biomarkers for research (Table 3).

**Table 3:** Expression levels of m6A modified bioactive substances in cancer exosomes

Cancer types	Biomarkers	Expression levels of biomarkers in exosomes from cancer patients	Main sources of evidence	References
ESCC	MiR-320b	Upregulated	Retrospective cohort studies	[106]
	NAT10	Upregulated	Retrospective cohort studies	[125]
GC	MiRNA-17-92	Upregulated	In vitro, animal models, retrospective cohort studies	[107]
LSCC	TUC338	Upregulated	In vitro	[116]
LUAD	MiR-3153	Upregulated	In vitro, animal models, retrospective cohort studies	[109]
	KLF6	Downregulated	Retrospective cohort studies	[126]
LC	MiR-125a-5p	Upregulated	In vitro, retrospective cohort studies	[108]
NSCLC	CircPACRGL	Upregulated	In vitro, retrospective cohort studies	[120]
GBC	TRPM2-AS	Upregulated	Retrospective cohort studies	[112]
BC	LINC 00657	Upregulated	Retrospective cohort studies	[114]
PCa	A1BG-AS1	Downregulated	Retrospective cohort studies	[117]
OSCC	FLG-AS1	Downregulated	Retrospective and prospective cohort studies	[115]
HBV-HCC	MAAS	Upregulated	Retrospective and prospective cohort studies	[110]
HCC	SLC16A1-AS1	Upregulated	prospective cohort studies	[111]
	CircCCAR1	Upregulated	In vitro, retrospective cohort	[118]
	Circ-CDYL	Upregulated	Retrospective cohort studies	[119]
	GPNMB	Upregulated	Retrospective cohort studies	[122]
CCa	Mortalin	Upregulated	Retrospective cohort studies	[123]
CRC	STING	Upregulated	Retrospective cohort studies	[124]
BCa	CircSLC38A1	Upregulated	In vitro, retrospective cohort studies	[121]
MM	LOC606724、	Upregulated	In vitro	[113]
	SNHG1			

*ESCC* esophageal squamous cell carcinoma, *GC* gastric cancer, *LSCC* laryngeal squamous cell carcinoma, *LUAD* lung adenocarcinoma, *LC* lung cancer, *NSCLC* non-small cell lung cancer, *GBC* gallbladder cancer, *BC* breast cancer, *PCa* prostate cancer, *OSCC* oral squamous cell carcinoma, *HBV-related HCC* hepatitis B virus-related hepatocellular carcinoma, *CCa* Cervical cancer, *CRC* colorectal cancer, *BCa* bladder cancer, *MM* multiple myeloma

However, it should be noted that the majority of the evidence listed in Table 3 comes from in vitro cell models, animal experiments, or small-scale retrospective clinical sample analysis, which belong to the early stages of discovery and validation. For example, METTL3 mediated m6A modification promotes the maturation of miR-320b and exosome packaging in ESCC. Retrospective analysis showed that elevated serum exosomal miR-320b levels in patients were associated with lymph node metastasis, suggesting its potential as a diagnostic tool, but further prospective validation is needed [106]. Similarly, m6A regulated circSLC38A1 is enriched in the serum exocrine of BCa patients, showing diagnostic value in a preliminary clinical study [121]. These are typical examples of early translational research. To transform the candidate molecules listed in Table 3 into clinically applicable diagnostic tools, it is not as simple as proving their expression differences, and a strict “validation funnel” must be traversed. Firstly, there is currently a lack of globally standardized procedures for the isolation and enrichment of exosome [138] and the specific detection of m6A modification [139–142]. The differences between different methods directly affect the accuracy and comparability of biomarker quantification across studies and centers, which is the first technical barrier to achieve clinical promotion. Secondly, candidate biomarkers must be demonstrated to have stable and reliable diagnostic sensitivity and specificity in a rigorously designed large, prospective, multicenter cohort, and establish clear clinical thresholds [143]. More importantly, intervention studies are needed to demonstrate that using this biomarker to guide clinical decision-making can ultimately improve patient outcomes and is cost-effective. Its value needs to go beyond existing standard methods [144].

## Targeting the M6A-exosome axis: bridging molecular mechanisms to clinical therapeutic translation

### The therapeutic potential of targeting the m6A-exosome axis

The m6A-exosome axis’s influence on the TME presents it as a possible therapeutic target for reducing tumor progression and increasing chemotherapy effectiveness. Since m6A methylation and demethylation are reversible processes, targeting m6A regulatory proteins to alter the expression of bioactive substances modified by m6A can exert antitumor therapeutic effects. Sinefungin, as the main inhibitor of METTL3, can reduce the levels of m6A modified miR-125a-5p in exosomes in vitro models, inhibit IGSF11 and its expression, thereby demonstrating the potential to enhance immune cell killing ability and inhibit lung cancer progression in a mechanistic manner [108]. Similarly, in preclinical models of multiple myeloma, knocking down the expression of METTL7A in adipocytes using specific small interfering RNA (siRNA) can reduce the levels of specific lncRNAs in exosomes, thereby enhancing the therapeutic effect of bortezomib on myeloma cells in vitro [113]. These studies provide principled evidence for METTL3 and METTL7A as therapeutic targets. In addition, there are clues indicating that m6A modification may influence exosome biogenesis, which comprises multivesicular bodies (MVBs) formation, cargo sorting, and MVBs fusion with the plasma membrane [54]. Zhou Jing and her research team found through in vitro and in vivo experiments that m6A modification impacts MVBs formation and fusion with the cell membrane, leading to a notable increase in exosome production and release. Conversely, inhibiting m6A methylation with 3-Deazaadenosine (DDA) and suppressing m6A-related enzymes can reduce exosome generation [145]. HnRNPA2/B1, a member of the reader proteins, plays a crucial role in the sorting of RNA into exosomes [35]. These findings collectively suggest that intervening in m6A modification not only regulates the generation of exosome cargo, but may also affect the production and delivery of exosome themselves, providing multiple potential intervention nodes for theoretically blocking the impact of this axis on the TME.

However, it should be emphasized that all the above evidence comes from preclinical studies (in vitro or animal models), and the mentioned compounds (such as sinefungin, DDA) or genetic tools (such as siRNA) are research tools used for mechanism exploration and have not undergone systematic clinical safety or toxicology evaluations, and cannot be directly equated with clinical candidate drugs. The importance of these studies lies in establishing the biological principles of the targets, but translating them into safe and effective therapies still faces significant challenges.

### Deep analysis of clinical translation of targeted therapy: the gap from mechanism concept to clinical drugs

The transformation of m6A-exosome axis as a therapeutic target is much more complex than proving its biological importance. The current research focuses on preclinical concept validation, while moving towards clinical practice faces a series of intertwined and severe substantive challenges, specifically reflected in the following aspects.

The fundamental contradiction between drug specificity and off target effects. The existing evidence heavily relies on non selective tool compounds. For example, sinefungin and DDA are broad-spectrum methylation inhibitors that not only inhibit METTL3, but also extensively interfere with other key methylation transfer processes within cells [146, 147]. This lack of selectivity means that while inhibiting tumor related pathways, it inevitably disrupts the epigenetic transcriptome homeostasis that normal cells rely on for survival, leading to unacceptable on target toxicity. Similarly, strategies targeting reading proteins such as hnRNPA2B1 also face serious off target risks due to their fundamental role in extensive RNA processing [38]. Therefore, the primary challenge at present is not to confirm the effectiveness of the target, but to design highly selective inhibitors that can accurately distinguish the function or structure of the target under “disease state” and “physiological state”.

Delivery challenges and implementation difficulties in in vivo pharmacodynamics. For nucleic acid based intervention strategies such as siMETTL7A, their clinical translation is still limited by the classic bottleneck of in vivo delivery. Exposed siRNA is easily degraded and difficult to efficiently and specifically enter target cells (such as tumor cells or specific stromal cells) [148]. Although technologies such as lipid nanoparticles have made breakthroughs in the field of vaccines, their application in systemic cancer treatment and achieving organ/cell specific targeting still faces multiple challenges such as stability, liver and spleen enrichment, immunogenicity, and penetration of solid tumor barriers [149]. How to accurately deliver therapeutic molecules to the target site of action and ensure their effective release within the cell is the key to determining the success or failure of such therapies.

Unresolved concerns about the toxicity safety of the system. Even if the issues of selectivity and delivery are addressed, the long-term systemic effects of intervening in m6A modification or exosome secretion, which are fundamental cellular biological processes, remain a huge unknown. Toxicological studies of preclinical animal models may not fully predict human reactions [150]. Potential risks include but are not limited to: immune system dysfunction, abnormal development of reproductive cells, increased risk of neurotoxicity and metabolic diseases [151–154]. Therefore, conducting comprehensive and long-term preclinical safety evaluations (including carcinogenicity, reproductive toxicity, etc.) is a mandatory and costly step in advancing any candidate drug into clinical practice.

The lack of regulatory pathways and the reality of clinical development stages. From the perspective of regulatory science, the development path of drugs with such novel mechanisms of action is complex. Early communication with regulatory agencies (such as FDA, NMPA) is required to determine a reasonable clinical pharmacology and toxicology research plan [155–157]. It is crucial to develop reliable pharmacodynamic biomarkers (such as dynamically monitoring changes in specific m6A markers in patient exosomes through liquid biopsy) to visually demonstrate in early clinical trials that drugs do indeed successfully act on predetermined targets (target engagement) in the human body.

The research on TME responsive nanoplatforms provides forward-looking ideas in addressing these challenges [158]. For example, manganese oxide-mesoporous carbon nanoparticles improve treatment accuracy and reduce systemic exposure by utilizing tumor specific microenvironment signals to achieve targeted controlled drug release. This suggests that future intervention strategies targeting the m6A-exosome axis may draw on similar “smart” delivery designs to deliver highly selective inhibitors or nucleic acid drugs specifically to the tumor site, thereby enhancing efficacy while minimizing off target toxicity. However, the most direct reflection of the current stage of transformation is that as of January 2026, there are no Phase I clinical trials of drugs directly targeting the core component of the “m6A-exosome axis” for cancer treatment. This blank starkly indicates that the field is still in the preclinical drug discovery and candidate compound optimization stage, and there is still a considerable distance to go from human trials. In contrast, some TME responsive nanoplatforms have accumulated more systematic biocompatibility, in vivo pharmacokinetics, and preliminary safety data, providing important evaluation frameworks and translational standards for preclinical development of novel therapies, including targeting m6A.

## Conclusions and perspectives

This review synthesizes evidence from 21 original preclinical studies published in the past five years and analyzes the impact of the “m6A-exosome axis” on the TME. The study elucidated that m6A modified bioactive substances are selectively packaged and delivered by exosome to receptor cells within the TME, thereby driving their systemic remodeling. Evidence suggests that this axis forms a crucial communication bridge between tumor cells and various matrix components through exosome mediated transmission. Among them, METTL3 and hnRNPA2B1, as regulatory factors that repeatedly appear in cancer, can coordinate the maturation process of key effector molecules and regulate the sorting mechanism of exosome. In addition, the included preclinical studies suggest that the most consistently TME hallmarks affected by the m6A-exosome axis is shaping the immunosuppressive phenotype by inducing polarization of M2 macrophages and depletion of T cells. This axis can also directly promote angiogenesis, EMT, metabolic reprogramming, and drug resistance, suggesting that it may promote tumor progression. It is worth noting that the biological effects of this axis have significant background dependence, and the same regulatory factor may play completely opposite roles in different cancer types and cellular environments.

In clinical significance, exosome carrying m6A modified biomolecules have shown potential as non-invasive liquid biopsy markers, and targeting this axis key node represents a promising preclinical treatment strategy. In addition to diagnostic potential, m6A modified exosome ncRNA has broad prospects as a novel therapeutic drug for reprogramming TME. Based on the mechanism summarized in this article, the following strategies can be envisaged. Firstly, targeting oncogenic ncRNAs in tumor derived exosomes can reverse immune suppression. For example, in GC, METTL3 dependent miR-17-92 cluster maturation and its exosome delivery can induce macrophage M2 polarization and the formation of an immunosuppressive microenvironment [107]; Therefore, the use of anti-miR-17-92 antisense oligonucleotides or miRNA sponges may block this process. Similarly, in HCC and NSCLC, exosome m6A modified lncRNAs SLC16A1-AS1 and circPACRGL promote M2 polarization, making them attractive therapeutic targets [111, 120]. Secondly, engineered exosomes can be used to deliver tumor suppressor ncRNA. For example, delivery of lncRNA FLG-AS1 in OSCC, delivery of lncRNA A1BG-AS1 in PCa, and delivery of KLF6 mRNA in LUAD all exhibit tumor suppressive function after exosome metastasis [115, 117, 126]. Thirdly, the combined use of m6A modified inhibitors and ncRNA therapy may produce synergistic anti-tumor effects. For example, in LC, the METTL3 inhibitor sinefungin can reduce the levels of m6A modified miR-125a-5p in exosome, thereby upregulating IGSF11 and enhancing immune cell killing ability [108]. In HCC, targeting FTO can downregulate GPNMB and restore CD8 + T cell function [122]. These strategies highlight the potential of ncRNA based interventions for precise regulation of immune cell function, angiogenesis, EMT, and drug resistance within the TME. However, it must be emphasized that all evidence in this article comes from preclinical models, and there are still multiple substantive challenges in understanding the mechanisms and clinical applications.

The current research has several limitations and provides directions for the future. Firstly, at the mechanistic level, the specific molecular mechanism by which m6A motifs guide the sorting of exosome is not yet clear and requires the use of more advanced techniques for analysis. Secondly, existing research has overly focused on RNA and a few proteins, and the roles of other m6A regulated goods such as metabolites and lipids urgently need to be explored. Furthermore, in order to enhance physiological relevance, it is necessary to strengthen validation in patient derived organoids, genetically engineered animal models, and clinical cohorts in the future. In terms of translational applications, developing candidate biomarkers into clinical tools urgently requires the standardization of exosome isolation and detection standards, and the validation of their efficacy through large-scale prospective studies. The transformation of treatment faces more severe challenges: the need to develop highly specific inhibitors to avoid interfering with normal physiological functions; Design efficient targeted delivery systems; Comprehensively evaluate the long-term safety of intervening in such basic cellular processes; And establish a clear clinical development pathway. In summary, the m6A-exosome axis establishes a new dimension of intercellular communication, enabling tumors to remotely epigenetic reprogram the TME. Deepening the understanding of this axis and innovatively addressing the bottleneck of its clinical translation is crucial for ultimately realizing its potential in precision diagnosis and treatment of tumors.

## Supplementary Information

Below is the link to the electronic supplementary material.

Supplementary Material 1.

## Data Availability

No datasets were generated or analysed during the current study.

## Abbreviations

TME: Tumor microenvironment
CAFs: Cancer associated fibroblasts
M6A: N6-methyladenosine
EMT: Epithelial-mesenchymal transition
ECM: Extracellular matrix
NcRNAs: Non-coding RNAs
MiRNAs: MicroRNAs
LncRNAs: Long non-coding RNAs
CircRNAs: Circular RNAs
PRISMA: Systematic reviews and meta-analyses
METTL3: Methyltransferase-like 3
WTAP: Wilms’ tumor 1-associated protein
RBM15: RNA-binding Motif protein 15
ZC3H13: Zinc finger CCCH-type containing 13
FTO: Fat mass and obesity-associated gene
ALKBH5: AlkB homolog 5
YTHDF: YTH domain family
IGF2BP1/2/3: Insulin-like growth factor 2 mRNA-binding proteins 1/2/3
elF3: eukaryotic initiation factor 3
HNRNP: Heterogeneous nuclear ribonucleoprotein
PD-L1: Programmed death-ligand 1
HCC: Hepatocellular carcinoma
LC: Lung cancer
BCa: Bladder cancer
CRC: Colorectal cancer
OS: Osteosarcoma
PMN: Pre-metastatic Niche
BR: Butyrate
IL-8: Interleukin-8
FGF: Fibroblast growth factor
VEGF: Vascular endothelial growth factor
TGF-β: Transforming growth factor-beta
RISC: RNA-induced silencing complexes
Pri-miRNAs: Primary miRNAs
DGCR8: DiGeorge cirtical region 8
Pre-miRNAs: Precursor miRNAs
GC: Gastric cancer
LUAD: Lung adenocarcinoma
IGSF11: Immunoglobulin superfamily member 11
ESCC: Esophageal squamous cell carcinoma
OSCC: Oral squamous cell carcinoma
GPNMB: Glycoprotein non-metastatic melanoma protein B
STING: Interferon gene exosome stimulator
NAT10: N-acetyltransferase 10
KLF6: Krüppel-like factor 6
MVBs: Multivesicular bodies
SiRNA: Small interfering RNA
DDA: 3-Deazaadenosine

## References

[1] Xiao Y, Yu D. Tumor microenvironment as a therapeutic target in cancer. Pharmacol Ther. 2021;221:107753. 10.1016/j.pharmthera.2020.107753. 33259885 10.1016/j.pharmthera.2020.107753 PMC8084948

[2] Whiteside TL. The tumor microenvironment and its role in promoting tumor growth. Oncogene. 2008;27(45):5904–12. 18836471 10.1038/onc.2008.271 PMC3689267

[3] Zhang Q, Wang W, Zhou Q, Chen C, Yuan W, Liu J, Li X, Sun Z. Roles of circRNAs in the tumour microenvironment. Mol Cancer. 2020;19(1):14. 10.1186/s12943-019-1125-9. 31973726 10.1186/s12943-019-1125-9 PMC6977266

[4] Wang D, Han Y, Peng L, Huang T, He X, Wang J, Ou C. Crosstalk between N6-methyladenosine (m6A) modification and noncoding RNA in tumor microenvironment. Int J Biol Sci. 2023;19(7):2198–219. 10.7150/ijbs.79651. 37151887 10.7150/ijbs.79651 PMC10158024

[5] Cantara WA, Crain PF, Rozenski J, McCloskey JA, Harris KA, Zhang X, Vendeix FA, Fabris D, Agris PF. The RNA Modification Database, RNAMDB: 2011 update. Nucleic Acids Res. 2011;39(Database issue):D195–201. 10.1093/nar/gkq1028. 21071406 10.1093/nar/gkq1028 PMC3013656

[6] Meyer KD, Saletore Y, Zumbo P, Elemento O, Mason CE, Jaffrey SR. Comprehensive analysis of mRNA methylation reveals enrichment in 3’ UTRs and near stop codons. Cell. 2012;149(7):1635–46. . 22608085 10.1016/j.cell.2012.05.003 PMC3383396

[7] Dominissini D, Moshitch-Moshkovitz S, Schwartz S, Salmon-Divon M, Ungar L, Osenberg S, Cesarkas K, Jacob-Hirsch J, Amariglio N, Kupiec M, Sorek R, Rechavi G. Topology of the human and mouse m6A RNA methylomes revealed by m6A-seq. Nature. 2012;485(7397):201–6. 10.1038/nature11112. 22575960 10.1038/nature11112

[8] An Y, Duan H. The role of m6A RNA methylation in cancer metabolism. Mol Cancer. 2022;21(1):14. 10.1186/s12943-022-01500-4. 35022030 10.1186/s12943-022-01500-4 PMC8753874

[9] Ma S, Chen C, Ji X, Liu J, Zhou Q, Wang G, Yuan W, Kan Q, Sun Z. The interplay between m6A RNA methylation and noncoding RNA in cancer. J Hematol Oncol. 2019;12(1):121. 10.1186/s13045-019-0805-7. 31757221 10.1186/s13045-019-0805-7 PMC6874823

[10] Kalluri R, LeBleu VS. The biology, function, and biomedical applications of exosomes. Science. 2020;367(6478). 10.1126/science.aau6977 10.1126/science.aau6977 PMC7717626 32029601

[11] Yang D, Zhang W, Zhang H, Zhang F, Chen L, Ma L, Larcher LM, Chen S, Liu N, Zhao Q, Tran PHL, Chen C, Veedu RN, Wang T. Progress, opportunity, and perspective on exosome isolation - efforts for efficient exosome-based theranostics. Theranostics. 2020;10(8):3684–707. 10.7150/thno.41580. 32206116 10.7150/thno.41580 PMC7069071

[12] Tenchov R, Sasso JM, Wang X, Liaw WS, Chen CA, Zhou QA. ExosomesNature’s lipid nanoparticles, a rising star in drug delivery and diagnostics. ACS Nano. 2022;16(11):17802–46. 10.1021/acsnano.2c08774. 36354238 10.1021/acsnano.2c08774 PMC9706680

[13] Zhang Y, Liu Y, Liu H, Tang WH. Exosomes: biogenesis, biologic function and clinical potential. Cell Biosci. 2019;9:19. 10.1186/s13578-019-0282-2. 30815248 10.1186/s13578-019-0282-2 PMC6377728

[14] Gurung S, Perocheau D, Touramanidou L, Baruteau J. The exosome journey: from biogenesis to uptake and intracellular signalling. Cell Commun Signal. 2021;19(1):47. 10.1186/s12964-021-00730-1. 33892745 10.1186/s12964-021-00730-1 PMC8063428

[15] Qi YK, Zheng JS, Liu L. Mirror-image protein and peptide drug discovery through mirror-image phage display. CHEM. 2024;10(8):2390–407. 10.1016/j.chempr.2024.06.004.

[16] Fu XY, Yin H, Chen XT, Yao JF, Ma YN, Song M, Xu H, Yu QY, Du SS, Qi YK, Wang KW. Three rounds of stability-guided optimization and systematical evaluation of oncolytic peptide LTX-315. 2024;67(5):3885–908 10.1021/acs.jmedchem.3c02232 10.1021/acs.jmedchem.3c02232 38278140

[17] Rethlefsen ML, Page MJ. PRISMA 2020 and PRISMA-S: common questions on tracking records and the flow diagram. J Med Libr Assoc. 2022;110(2):253–7. 10.5195/jmla.2022.1449. 35440907 10.5195/jmla.2022.1449 PMC9014944

[18] Hooijmans CR, Rovers MM, de Vries RB, Leenaars M, Ritskes-Hoitinga M, Langendam MW. SYRCLE’s risk of bias tool for animal studies. BMC Med Res Methodol. 2014;14:43. 10.1186/1471-2288-14-43. 24667063 10.1186/1471-2288-14-43 PMC4230647

[19] Pan S, Deng Y, Fu J, Zhang Y, Zhang Z, Qin X. N6–methyladenosine upregulates miR–181d–5p in exosomes derived from cancer–associated fibroblasts to inhibit 5–FU sensitivity by targeting NCALD in colorectal cancer. Int J Oncol. 2022. 10.3892/ijo.2022.5304 10.3892/ijo.2022.5304 PMC8759347 35014676

[20] Huang W, Chen TQ, Fang K, Zeng ZC, Ye H, Chen YQ. N6-methyladenosine methyltransferases: functions, regulation, and clinical potential. J Hematol Oncol. 2021;14(1):117. 10.1186/s13045-021-01129-8. 34315512 10.1186/s13045-021-01129-8 PMC8313886

[21] Deng X, Su R, Weng H, Huang H, Li Z, Chen J. RNA N(6)-methyladenosine modification in cancers: current status and perspectives. Cell Res. 2018;28(5):507–17. 10.1038/s41422-018-0034-6. 29686311 10.1038/s41422-018-0034-6 PMC5951805

[22] Panneerdoss S, Eedunuri VK, Yadav P, Timilsina S, Rajamanickam S, Viswanadhapalli S, Abdelfattah N, Onyeagucha BC, Cui X, Lai Z, Mohammad TA, Gupta YK, Huang TH, Huang Y, Chen Y, Rao MK. Cross-talk among writers, readers, and erasers of m(6)A regulates cancer growth and progression. Sci Adv. 2018;4(10):eaar8263. 10.1126/sciadv. 30306128 10.1126/sciadv.aar8263 PMC6170038

[23] Liu J, Yue Y, Han D, Wang X, Fu Y, Zhang L, Jia G, Yu M, Lu Z, Deng X, Dai Q, Chen W, He C. A METTL3-METTL14 complex mediates mammalian nuclear RNA N6-adenosine methylation. Nat Chem Biol. 2014;10(2):93–5. 10.1038/nchembio.1432. 24316715 10.1038/nchembio.1432 PMC3911877

[24] Russell DA, Chau MK, Shi Y, Levasseur IN, Maldonato BJ, Totah RA. METTL7A (TMT1A) and METTL7B (TMT1B) are responsible for Alkyl S-thiol methyl transferase activity in liver. Drug Metab Dispos. 2023;51(8):1024–34. 10.1124/dmd.123.001268. 37137720 10.1124/dmd.123.001268 PMC10353073

[25] Patil DP, Chen CK, Pickering BF, Chow A, Jackson C, Guttman M, Jaffrey SR. m(6)A RNA methylation promotes XIST-mediated transcriptional repression. Nature. 2016;537(7620):369–73. 10.1038/nature19342. 27602518 10.1038/nature19342 PMC5509218

[26] Wen J, Lv R, Ma H, Shen H, He C, Wang J, Jiao F, Liu H, Yang P, Tan L, Lan F, Shi YG, He C, Shi Y, Diao J. Zc3h13 Regulates nuclear RNA m(6)A methylation and mouse embryonic stem cell self-renewal. Mol Cell. 2018;69(6):1028–38. 10.1016/j.molcel.2018.02.015. 29547716 10.1016/j.molcel.2018.02.015 PMC5858226

[27] Jia G, Fu Y, Zhao X, Dai Q, Zheng G, Yang Y, Yi C, Lindahl T, Pan T, Yang YG, He C. N6-methyladenosine in nuclear RNA is a major substrate of the obesity-associated FTO. Nat Chem Biol. 2011;7(12):885–7. 10.1038/nchembio.687. 22002720 10.1038/nchembio.687 PMC3218240

[28] Xu C, Liu K, Tempel W, Demetriades M, Aik W, Schofield CJ, Min J. Structures of human ALKBH5 demethylase reveal a unique binding mode for specific single-stranded N6-methyladenosine RNA demethylation. J Biol Chem. 2014;289(25):17299–311. 10.1074/jbc.M114.550350. 24778178 10.1074/jbc.M114.550350 PMC4067165

[29] Huang H, Weng H, Sun W, Qin X, Shi H, Wu H, Zhao BS, Mesquita A, Liu C, Yuan CL, Hu YC, Hüttelmaier S, Skibbe JR, Su R, Deng X, Dong L, Sun M, Li C, Nachtergaele S, Wang Y, Hu C, Ferchen K, Greis KD, Jiang X, Wei M, Qu L, Guan JL, He C, Yang J, Chen J. Recognition of RNA N(6)-methyladenosine by IGF2BP proteins enhances mRNA stability and translation. Nat Cell Biol. 2018;20(3):285–95. 10.1038/s41556-018-0045-z. 29476152 10.1038/s41556-018-0045-z PMC5826585

[30] Zhou J, Wan J, Gao X, Zhang X, Jaffrey SR, Qian SB. Dynamic m(6)A mRNA methylation directs translational control of heat shock response. Nature. 2015;526(7574):591–4. 10.1038/nature15377. 26458103 10.1038/nature15377 PMC4851248

[31] Li Z, Zhang J, Liu X, Li S, Wang Q, Di C, Hu Z, Yu T, Ding J, Li J, Yao M, Fan J, Huang S, Gao Q, Zhao Y, He X. The LINC01138 drives malignancies via activating arginine methyltransferase 5 in hepatocellular carcinoma. Nat Commun. 2018;9(1):1572. 10.1038/s41467-018-04006-0. 29679004 10.1038/s41467-018-04006-0 PMC5910401

[32] Xiao W, Adhikari S, Dahal U, Chen YS, Hao YJ, Sun BF, Sun HY, Li A, Ping XL, Lai WY, Wang X, Ma HL, Huang CM, Yang Y, Huang N, Jiang GB, Wang HL, Zhou Q, Wang XJ, Zhao YL, Yang YG. Nuclear m(6)A Reader YTHDC1 Regulates mRNA. Splicing Mol Cell. 2016;61(4):507–19. 10.1016/j.molcel.2016.01.012. 26876937 10.1016/j.molcel.2016.01.012

[33] Hsu PJ, Zhu Y, Ma H, Guo Y, Shi X, Liu Y, Qi M, Lu Z, Shi H, Wang J, Cheng Y, Luo G, Dai Q, Liu M, Guo X, Sha J, Shen B, He C. Ythdc2 is an N(6)-methyladenosine binding protein that regulates mammalian spermatogenesis. Cell Res. 2017;27(9):1115–27. 10.1038/cr.2017.99. 28809393 10.1038/cr.2017.99 PMC5587856

[34] Liu N, Dai Q, Zheng G, He C, Parisien M, Pan T. N(6)-methyladenosine-dependent RNA structural switches regulate RNA-protein interactions. Nature. 2015;518(7540):560–4. 10.1038/nature14234. 25719671 10.1038/nature14234 PMC4355918

[35] Alarcón CR, Goodarzi H, Lee H, Liu X, Tavazoie S, Tavazoie SF. HNRNPA2B1 is a mediator of m(6)A-dependent nuclear RNA processing events. Cell. 2015;162(6):1299–308. 10.1016/j.cell.2015.08.011 26321680 10.1016/j.cell.2015.08.011 PMC4673968

[36] Meyer KD, Patil DP, Zhou J, Zinoviev A, Skabkin MA, Elemento O, Pestova TV, Qian SB, Jaffrey SR. 5’ UTR m(6)A promotes cap-independent translation. Cell. 2015;163(4):999–1010. 10.1016/j.cell.2015.10.012 26593424 10.1016/j.cell.2015.10.012 PMC4695625

[37] Cai Z, Xu H, Bai G, Hu H, Wang D, Li H, Wang Z. ELAVL1 promotes prostate cancer progression by interacting with other m6A regulators. Front Oncol. 2022;12:939784. 10.3389/fonc.2022.939784. 35978821 10.3389/fonc.2022.939784 PMC9376624

[38] Wang T, Kong S, Tao M, Ju S. The potential role of RNA N6-methyladenosine in Cancer progression. Mol Cancer. 2020;19(1):88. 10.1186/s12943-020-01204-7. 32398132 10.1186/s12943-020-01204-7 PMC7216508

[39] Weng H, Huang H, Wu H, Qin X, Zhao BS, Dong L, Shi H, Skibbe J, Shen C, Hu C, Sheng Y, Wang Y, Wunderlich M, Zhang B, Dore LC, Su R, Deng X, Ferchen K, Li C, Sun M, Lu Z, Jiang X, Marcucci G, Mulloy JC, Yang J, Qian Z, Wei M, He C, Chen J. METTL14 Inhibits Hematopoietic Stem/Progenitor Differentiation and Promotes Leukemogenesis via mRNA m(6)A Modification. Cell Stem Cell. 2018;22(2):191–205. 10.1016/j.stem.2017.11.016. 29290617 10.1016/j.stem.2017.11.016 PMC5860916

[40] Chen M, Wei L, Law CT, Tsang FH, Shen J, Cheng CL, Tsang LH, Ho DW, Chiu DK, Lee JM, Wong CC, Ng IO, Wong CM. RNA N6-methyladenosine methyltransferase-like 3 promotes liver cancer progression through YTHDF2-dependent posttranscriptional silencing of SOCS2. Hepatology. 2018;67(6):2254–70. 10.1002/hep.29683 10.1002/hep.29683 29171881

[41] Xu J, Wan Z, Tang M, Lin Z, Jiang S, Ji L, Gorshkov K, Mao Q, Xia S, Cen D, Zheng J, Liang X, Cai X. N(6)-methyladenosine-modified CircRNA-SORE sustains sorafenib resistance in hepatocellular carcinoma by regulating β-catenin signaling. Mol Cancer. 2020;19(1):163. 10.1186/s12943-020-01281-8. 33222692 10.1186/s12943-020-01281-8 PMC7681956

[42] Niu Y, Lin Z, Wan A, Chen H, Liang H, Sun L, Wang Y, Li X, Xiong XF, Wei B, Wu X, Wan G. RNA N6-methyladenosine demethylase FTO promotes breast tumor progression through inhibiting BNIP3. Mol Cancer. 2019;18(1):46. 10.1186/s12943-019-1004-4. 30922314 10.1186/s12943-019-1004-4 PMC6437932

[43] Wang B, Wang Y, Wang W, Wang Z, Zhang Y, Pan X, Wen X, Leng H, Guo J, Ma XX. WTAP/IGF2BP3 mediated m6A modification of the EGR1/PTEN axis regulates the malignant phenotypes of endometrial cancer stem cells. J Exp Clin Cancer Res. 2024;43(1):204. 10.1186/s13046-024-03120-w. 39044249 10.1186/s13046-024-03120-w PMC11264439

[44] Chen S, Zhou L, Wang Y. ALKBH5-mediated m(6)A demethylation of lncRNA PVT1 plays an oncogenic role in osteosarcoma. Cancer Cell Int. 2020;20:34. 10.1186/s12935-020-1105-6. 32021563 10.1186/s12935-020-1105-6 PMC6993345

[45] Yang X, Zhang S, He C, Xue P, Zhang L, He Z, Zang L, Feng B, Sun J, Zheng M. METTL14 suppresses proliferation and metastasis of colorectal cancer by down-regulating oncogenic long non-coding RNA XIST. Mol Cancer. 2020;19(1):46. 10.1186/s12943-020-1146-4. 32111213 10.1186/s12943-020-1146-4 PMC7047419

[46] Guo Y, Guo Y, Chen C, Fan D, Wu X, Zhao L, Shao B, Sun Z, Ji Z. Circ3823 contributes to growth, metastasis and angiogenesis of colorectal cancer: involvement of miR-30c-5p/TCF7 axis. Mol Cancer. 2021;20(1):93. 10.1186/s12943-021-01372-0. 34172072 10.1186/s12943-021-01372-0 PMC8229759

[47] Zhu X, Zhang P. m6A-modified circXPO1 accelerates colorectal cancer progression via interaction with FMRP to promote WWC2 mRNA decay. J Transl Med. 2024;22(1):931. 10.1186/s12967-024-05716-4. 39402642 10.1186/s12967-024-05716-4 PMC11472528

[48] Liu Z, Wang T, She Y, Wu K, Gu S, Li L, Dong C, Chen C, Zhou Y. N(6)-methyladenosine-modified circIGF2BP3 inhibits CD8(+) T-cell responses to facilitate tumor immune evasion by promoting the deubiquitination of PD-L1 in non-small cell lung cancer. Mol Cancer. 2021;20(1):105. 10.1186/s12943-021-01398-4. 34416901 10.1186/s12943-021-01398-4 PMC8377850

[49] Wang R, Ye H, Yang B, Ao M, Yu X, Wu Y, Xi M, Hou M. m6A-modified circNFIX promotes ovarian cancer progression and immune escape via activating IL-6R/JAK1/STAT3 signaling by sponging miR-647. Int Immunopharmacol. 2023;124(Pt A):110879. 10.1016/j.intimp.2023.110879. 37713785 10.1016/j.intimp.2023.110879

[50] Han J, Wang JZ, Yang X, Yu H, Zhou R, Lu HC, Yuan WB, Lu JC, Zhou ZJ, Lu Q, Wei JF, Yang H. METTL3 promote tumor proliferation of bladder cancer by accelerating pri-miR221/222 maturation in m6A-dependent manner. Mol Cancer. 2019;18(1):110. 10.1186/s12943-019-1036-9. 31228940 10.1186/s12943-019-1036-9 PMC6588935

[51] Borriello L, Seeger RC, Asgharzadeh S, DeClerck YA. More than the genes, the tumor microenvironment in neuroblastoma. Cancer Lett. 2016;380(1):304–14. 10.1016/j.canlet.2015.11.017. 26597947 10.1016/j.canlet.2015.11.017 PMC5558454

[52] Marques P, Grossman AB, Korbonits M. The tumour microenvironment of pituitary neuroendocrine tumours. Front Neuroendocrinol. 2020;58:100852. 10.1016/j.yfrne.2020.100852. 32553750 10.1016/j.yfrne.2020.100852

[53] Zhang L, Yu D. Exosomes in cancer development, metastasis, and immunity. Biochim Biophys. Acta Rev Cancer. 2019;1871(2):455–68. 10.1016/j.bbcan.2019.04.004. 31047959 10.1016/j.bbcan.2019.04.004 PMC6542596

[54] Théry C, Zitvogel L, Amigorena S. Exosomes: composition, biogenesis and function. Nat Rev Immunol. 2002;2(8):569–79. 10.1038/nri855. 12154376 10.1038/nri855

[55] Aakel N, Mohammed R, Fathima A, Kerzabi R, Abdallah A, Ibrahim WN. Role of exosome in solid cancer progression and its potential therapeutics in cancer treatment. Cancer Med. 2025;14(9):e70941. 10.1002/cam4.70941. 40344389 10.1002/cam4.70941 PMC12063069

[56] Liu M, Wen Z, Zhang T, Zhang L, Liu X, Wang M. The role of exosomal molecular cargo in exosome biogenesis and disease diagnosis. Front Immunol. 2024;15:1417758. 10.3389/fimmu.2024.1417758. 38983854 10.3389/fimmu.2024.1417758 PMC11231912

[57] Yang E, Wang X, Gong Z, Yu M, Wu H, Zhang D. Exosome-mediated metabolic reprogramming: the emerging role in tumor microenvironment remodeling and its influence on cancer progression. Signal Transduct Target Ther. 2020;5(1):242. 10.1038/s41392-020-00359-5. 33077737 10.1038/s41392-020-00359-5 PMC7572387

[58] Liu J, Ren L, Li S, Li W, Zheng X, Yang Y, Fu W, Yi J, Wang J, Du G. The biology, function, and applications of exosomes in cancer. Acta Pharm Sin B. 2021;11(9):2783–97. 10.1016/j.apsb.2021.01.001. 34589397 10.1016/j.apsb.2021.01.001 PMC8463268

[59] Kaczmarek JC, Kowalski PS, Anderson DG. Advances in the delivery of RNA therapeutics: from concept to clinical reality. Genome Med. 2017;9(1):60. 10.1186/s13073-017-0450-0. 28655327 10.1186/s13073-017-0450-0 PMC5485616

[60] Iqbal Z, Rehman K, Mahmood A, Shabbir M, Liang Y, Duan L, Zeng H. Exosome for mRNA delivery: strategies and therapeutic applications. J Nanobiotechnol. 2024;22(1):395. 10.1186/s12951-024-02634-x. 10.1186/s12951-024-02634-x PMC11225225 38965553

[61] Soldevilla B, Rodríguez M, San Millán C, García V, Fernández-Periañez R, Gil-Calderón B, Martín P, García-Grande A, Silva J, Bonilla F, Domínguez G. Tumor-derived exosomes are enriched in ∆Np73, which promotes oncogenic potential in acceptor cells and correlates with patient survival. Hum Mol Genet. 2014;23(2):467–78. 10.1093/hmg/ddt437. 24067531 10.1093/hmg/ddt437

[62] Fatima F, Nawaz M. Stem cell-derived exosomes: roles in stromal remodeling, tumor progression, and cancer immunotherapy. Chin J Cancer. 2015;34(12):541–53. 10.1186/s40880-015-0051-5. 26369565 10.1186/s40880-015-0051-5 PMC4593342

[63] Dou Q, Wang J, Yang Y, Zhuo W. Roles of exosome-derived non-coding RNA in tumor micro-environment and its clinical application. Zhejiang Da Xue Xue Bao Yi Xue Ban. 2023;52(4):429–38. 10.3724/zdxbyxb-2023-0056. 37643977 10.3724/zdxbyxb-2023-0056 PMC10495245

[64] Li Y, Zhao Z, Liu W, Li X. SNHG3 Functions as miRNA Sponge to Promote Breast Cancer Cells Growth Through the Metabolic Reprogramming. Appl Biochem Biotechnol. 2020;191(3):1084–99. 10.1007/s12010-020-03244-7. 31956955 10.1007/s12010-020-03244-7 PMC7320061

[65] Chen S, Chen X, Luo Q, Liu X, Wang X, Cui Z, He A, He S, Jiang Z, Wu N, Chen P, Yu K, Zhuang J. Retinoblastoma cell-derived exosomes promote angiogenesis of human vesicle endothelial cells through microRNA-92a-3p. Cell Death Dis. 2021;12(7):695. 10.1038/s41419-021-03986-0. 34257272 10.1038/s41419-021-03986-0 PMC8277798

[66] Grelet S, Link LA, Howley B, Obellianne C, Palanisamy V, Gangaraju VK, Diehl JA, Howe PH. A regulated PNUTS mRNA to lncRNA splice switch mediates EMT and tumour progression. Nat Cell Biol. 2017;19(9):1105–15. 10.1038/ncb3595. 28825698 10.1038/ncb3595 PMC5578890

[67] Hwang S, Yang YM. Exosomal microRNAs as diagnostic and therapeutic biomarkers in non-malignant liver diseases. Arch Pharm Res. 2021;44(6):574–87. 10.1007/s12272-021-01338-2. 34165701 10.1007/s12272-021-01338-2 PMC8223764

[68] Rana S, Malinowska K, Zöller M. Exosomal tumor microRNA modulates premetastatic organ cells. Neoplasia. 2013;15(3):281–95. 10.1593/neo.122010. 23479506 10.1593/neo.122010 PMC3593151

[69] Wen B, Tao R, Liu Y, Zhang Z. Investigating the role of exosomal microRNA-5703 in modulating tumor-associated endothelial cells in lung cancer. Cytojournal. 2024;21:77. 10.25259/Cytojournal_99_2024. 39917007 10.25259/Cytojournal_99_2024 PMC11801689

[70] Luo G, Zhou Z, Cao Z, Huang C, Li C, Li X, Deng C, Wu P, Yang Z, Tang J, Qing L. M2 macrophage-derived exosomes induce angiogenesis and increase skin flap survival through HIF1AN/HIF-1α/VEGFA control. Arch Biochem Biophys. 2024;751:109822. 10.1016/j.abb.2023.109822. 38030054 10.1016/j.abb.2023.109822

[71] Li C, Ni YQ, Xu H, Xiang QY, Zhao Y, Zhan JK, He JY, Li S, Liu YS. Roles and mechanisms of exosomal non-coding RNAs in human health and diseases. Signal Transduct Target Ther. 2021;6(1):383. 10.1038/s41392-021-00779-x. 34753929 10.1038/s41392-021-00779-x PMC8578673

[72] Guo X, Qiu W, Liu Q, Qian M, Wang S, Zhang Z, Gao X, Chen Z, Xue H, Li G. Immunosuppressive effects of hypoxia-induced glioma exosomes through myeloid-derived suppressor cells via the miR-10a/Rora and miR-21/Pten Pathways. Oncogene. 2018;37(31):4239–59. 10.1038/s41388-018-0261-9. 29713056 10.1038/s41388-018-0261-9

[73] Cooks T, Pateras IS, Jenkins LM, Patel KM, Robles AI, Morris J, Forshew T, Appella E, Gorgoulis VG, Harris CC. Mutant p53 cancers reprogram macrophages to tumor supporting macrophages via exosomal miR-1246. Nat Commun. 2018;9(1):771. 10.1038/s41467-018-03224-w. 29472616 10.1038/s41467-018-03224-w PMC5823939

[74] Smolle MA, Prinz F, Calin GA, Pichler M. Current concepts of non-coding RNA regulation of immune checkpoints in cancer. Mol Aspects Med. 2019;70:117–26. 10.1016/j.mam.2019.09.007. 31582259 10.1016/j.mam.2019.09.007

[75] Oztatlici M, Ozdemir A, Oztatlici H, Kucukhuyuk S, Ozgul Ozdemir R, Orhan H, Ozbilgin K. Immunomodulatory effects of MDA-MB-231-derived exosome mimetic nanovesicles on CD4 + T cell line. Eurasian J Med Oncol. 2024. 10.14744/ejmo.2024.64067

[76] Lv K, Zhang Y, Yin G, Li X, Zhong M, Zhu X, Pei W. Extracellular vesicles derived from lung M2 macrophages enhance group 2 innate lymphoid cells function in allergic airway inflammation. Exp Mol Med. 2025;57(6):1202–15. 10.1038/s12276-025-01465-6. 40451928 10.1038/s12276-025-01465-6 PMC12229531

[77] Entezari M, Ghanbarirad M, Taheriazam A, Sadrkhanloo M, Zabolian A, Goharrizi M, Hushmandi K, Aref AR, Ashrafizadeh M, Zarrabi A, Nabavi N, Rabiee N, Hashemi M, Samarghandian S. Long non-coding RNAs and exosomal lncRNAs: potential functions in lung cancer progression, drug resistance and tumor microenvironment remodeling. Biomed Pharmacother. 2022;150:112963. 10.1016/j.biopha.2022.112963. 35468579 10.1016/j.biopha.2022.112963

[78] Xie H, Yao J, Wang Y, Ni B. Exosome-transmitted circVMP1 facilitates the progression and cisplatin resistance of non-small cell lung cancer by targeting miR-524-5p-METTL3/SOX2 axis. Drug Deliv. 2022;29(1):1257–71. 10.1080/10717544.2022.2057617. 35467477 10.1080/10717544.2022.2057617 PMC9045767

[79] Wang X, Huang J, Chen W, Li G, Li Z, Lei J. The updated role of exosomal proteins in the diagnosis, prognosis, and treatment of cancer. Exp Mol Med. 2022;54(9):1390–400. 10.1038/s12276-022-00855-4. 36138197 10.1038/s12276-022-00855-4 PMC9535014

[80] Maji S, Chaudhary P, Akopova I, Nguyen PM, Hare RJ, Gryczynski I, Vishwanatha JK. Exosomal annexin II promotes angiogenesis and breast cancer metastasis. Mol Cancer Res. 2017;15(1):93–105. 10.1158/1541-7786.Mcr-16-0163. 27760843 10.1158/1541-7786.MCR-16-0163 PMC5215956

[81] Horie K, Kawakami K, Fujita Y, Sugaya M, Kameyama K, Mizutani K, Deguchi T, Ito M. Exosomes expressing carbonic anhydrase 9 promote angiogenesis. Biochem Biophys Res Commun. 2017;492(3):356–61. 10.1016/j.bbrc.2017.08.107. 28851650 10.1016/j.bbrc.2017.08.107

[82] Huang Z, Yang M, Li Y, Yang F, Feng Y. Exosomes Derived from Hypoxic Colorectal Cancer Cells Transfer Wnt4 to Normoxic Cells to Elicit a Prometastatic Phenotype. Int J Biol Sci. 2018;14(14):2094–102. 10.7150/ijbs.28288. 30585272 10.7150/ijbs.28288 PMC6299371

[83] Hicklin DJ, Ellis LM. Role of the vascular endothelial growth factor pathway in tumor growth and angiogenesis. J Clin Oncol. 2005;23(5):1011–27. 10.1200/jco.2005.06.081. 15585754 10.1200/JCO.2005.06.081

[84] Dhar R, Devi A, Gorai S, Jha SK, Alexiou A, Papadakis M. Exosome and epithelial-mesenchymal transition: A complex secret of cancer progression. J Cell Mol Med. 2023;27(11):1603–7. 10.1111/jcmm.17755. 37183560 10.1111/jcmm.17755 PMC10243158

[85] Geiger TR, Peeper DS. Metastasis mechanisms. Biochim Biophys Acta. 2009;1796(2):293–308. 10.1016/j.bbcan.2009.07.006. 19683560 10.1016/j.bbcan.2009.07.006

[86] Mashouri L, Yousefi H, Aref AR, Ahadi AM, Molaei F, Alahari SK. Exosomes: composition, biogenesis, and mechanisms in cancer metastasis and drug resistance. Mol Cancer. 2019;18(1):75. 10.1186/s12943-019-0991-5. 30940145 10.1186/s12943-019-0991-5 PMC6444571

[87] Kim HS, Kim HJ, Lee MR, Han I. EMMPRIN expression is associated with metastatic progression in osteosarcoma. BMC Cancer. 2021;21(1):1059. 10.1186/s12885-021-08774-9. 34565336 10.1186/s12885-021-08774-9 PMC8474954

[88] Del Re M, Marconcini R, Pasquini G, Rofi E, Vivaldi C, Bloise F, Restante G, Arrigoni E, Caparello C, Bianco MG, Crucitta S, Petrini I, Vasile E, Falcone A, Danesi R. PD-L1 mRNA expression in plasma-derived exosomes is associated with response to anti-PD-1 antibodies in melanoma and NSCLC. Br J Cancer. 2018;118(6):820–4. 10.1038/bjc.2018.9. 29509748 10.1038/bjc.2018.9 PMC5886129

[89] Chen J, Song Y, Miao F, Chen G, Zhu Y, Wu N, Pang L, Chen Z, Chen X. PDL1-positive exosomes suppress antitumor immunity by inducing tumor-specific CD8(+) T cell exhaustion during metastasis. Cancer Sci. 2021;112(9):3437–54. 10.1111/cas.15033. 34152672 10.1111/cas.15033 PMC8409314

[90] Nittayaboon K, Leetanaporn K, Sangkhathat S, Roytrakul S, Navakanitworakul R. Proteomic analysis of butyrate-resistant colorectal cancer-derived exosomes reveals potential resistance to anti-cancer drugs. Discov Med. 2024;36(185):1306–15. 10.24976/Discov.Med.202436185.121 38926117 10.24976/Discov.Med.202436185.121

[91] Tufail M, Wu C, Hussain MS. Dietary, addictive and habitual factors, and risk of colorectal cancer. Nutrition. 2024;120:112334. 10.1016/j.nut.2023.112334. 38271761 10.1016/j.nut.2023.112334

[92] Tan T, Li J, Luo R, Wang R, Yin L, Liu M, Zeng Y, Zeng Z, Xie T. Recent Advances in understanding the mechanisms of elemene in reversing drug resistance in tumor cells: a review. Molecules. 2021. 10.3390/molecules26195792 10.3390/molecules26195792 PMC8510449 34641334

[93] Pan Y, Lin Y, Mi C. Cisplatin-resistant osteosarcoma cell-derived exosomes confer cisplatin resistance to recipient cells in an exosomal circ_103801-dependent manner. Cell Biol Int. 2021;45(4):858–68. 10.1002/cbin.11532. 33325136 10.1002/cbin.11532

[94] Zhou J, Tan X, Tan Y, Li Q, Ma J, Wang G. Mesenchymal Stem Cell Derived Exosomes in Cancer Progression, Metastasis and Drug Delivery: A Comprehensive Review. J Cancer. 2018;9(17):3129–37. 10.7150/jca.25376. 30210636 10.7150/jca.25376 PMC6134817

[95] Qi R, Zhu G, Wang Y, Wu S, Li S, Zhang D, Bu Y, Bhave G, Han R, Liu X. Microfluidic device for the analysis of MDR cancerous cell-derived exosomes’ response to nanotherapy. Biomed Microdevices. 2019;21(2):35. 10.1007/s10544-019-0381-1. 30906967 10.1007/s10544-019-0381-1 PMC6532782

[96] Kalluri R, LeBleu VS. Discovery of Double-Stranded Genomic DNA in Circulating Exosomes. Cold Spring Harb Symp. Quant Biol. 2016;81:275–80. 10.1101/sqb.2016.81. 10.1101/sqb.2016.81.030932 28424339

[97] Takahashi A, Okada R, Nagao K, Kawamata Y, Hanyu A, Yoshimoto S, Takasugi M, Watanabe S, Kanemaki MT, Obuse C, Hara E. Exosomes maintain cellular homeostasis by excreting harmful DNA from cells. Nat Commun. 2017;8:15287. 10.1038/ncomms15287. 28508895 10.1038/ncomms15287 PMC5440838

[98] Katoh M. Therapeutics targeting angiogenesis: genetics and epigenetics, extracellular miRNAs and signaling networks (Review). Int J Mol Med. 2013;32(4):763–7. 10.3892/ijmm.2013.1444. 23863927 10.3892/ijmm.2013.1444 PMC3812243

[99] Nishida N, Yano H, Nishida T, Kamura T, Kojiro M. Angiogenesis in cancer. Vasc Health Risk Manag. 2006;2(3):213–9. 10.2147/vhrm.2006.2.3.213. 17326328 10.2147/vhrm.2006.2.3.213 PMC1993983

[100] Zhao H, Yang L, Baddour J, Achreja A, Bernard V, Moss T, Marini JC, Tudawe T, Seviour EG, San Lucas FA, Alvarez H, Gupta S, Maiti SN, Cooper L, Peehl D, Ram PT, Maitra A, Nagrath D. Tumor microenvironment derived exosomes pleiotropically modulate cancer cell metabolism. Elife. 2016;5:e10250. 10.7554/eLife.10250. 26920219 10.7554/eLife.10250 PMC4841778

[101] Achreja A, Zhao H, Yang L, Yun TH, Marini J, Nagrath D, Exo-MFA -. A 13 C metabolic flux analysis framework to dissect tumor microenvironment-secreted exosome contributions towards cancer cell metabolism. Metab Eng. 2017;43(Pt B):156–72. 10.1016/j.ymben.2017.01.001. 28087332 10.1016/j.ymben.2017.01.001 PMC7393794

[102] Iwakawa HO, Tomari Y. The Functions of MicroRNAs: mRNA Decay and Translational Repression. Trends Cell Biol. 2015;25(11):651–65. 10.1016/j.tcb.2015.07.011. 26437588 10.1016/j.tcb.2015.07.011

[103] Alarcón CR, Lee H, Goodarzi H, Halberg N, Tavazoie SF. N6-methyladenosine marks primary microRNAs for processing. Nature. 2015;519(7544):482–5. 10.1038/nature14281. 25799998 10.1038/nature14281 PMC4475635

[104] Treiber T, Treiber N, Meister G. Regulation of microRNA biogenesis and its crosstalk with other cellular pathways. Nat Rev Mol Cell Biol. 2019;20(1):5–20. 10.1038/s41580-018-0059-1. 30228348 10.1038/s41580-018-0059-1

[105] Pérez-Boza J, Boeckx A, Lion M, Dequiedt F, Struman I. hnRNPA2B1 inhibits the exosomal export of miR-503 in endothelial cells. Cell Mol Life Sci. 2020;77(21):4413–28. 10.1007/s00018-019-03425-6. 31894362 10.1007/s00018-019-03425-6 PMC11104873

[106] Liu T, Li P, Li J, Qi Q, Sun Z, Shi S, Xie Y, Liu S, Wang Y, Du L, Wang C. Exosomal and intracellular miR-320b promotes lymphatic metastasis in esophageal squamous cell carcinoma. Mol Ther Oncolytics. 2021;23:163–80. 10.1016/j.omto.2021.09.003. 34729394 10.1016/j.omto.2021.09.003 PMC8526502

[107] Li S, Zhou J, Wang S, Yang Q, Nie S, Ji C, Zhang X, Li S, Zhou X, Chu J, Wu X, Jiao J, Xu R, Xu Q, Huang M, Wang Q, Dou L, Hu Q, Jiang F, Dai X, Nan Z, Song X, Zhang D, Liu L. N(6)-methyladenosine-regulated exosome biogenesis orchestrates an immunosuppressive pre-metastatic niche in gastric cancer peritoneal metastasis. Cancer Commun. 2025;45(8):941–65. 10.1002/cac2.70034 40370322 10.1002/cac2.70034 PMC12365546

[108] Bougras-Cartron G, Nadaradjane A, Joalland MP, Lalier-Bretaudeau L, Raimbourg J, Cartron PF. Adenosine methylation level of miR-125a-5p promotes anti-PD-1 therapy escape through the regulation of IGSF11/VSIG3 expression. Cancers. 2023;15(12). 10.3390/cancers15123188 10.3390/cancers15123188 PMC10296663 37370798

[109] Xu L, Wang L, Yang R, Li T, Zhu X. Lung adenocarcinoma cell-derived exosomes promote M2 macrophage polarization through transmission of miR-3153 to activate the JNK signaling pathway. Hum Mol Genet. 2023;32(13):2162–76. 10.1093/hmg/ddad052. 37010098 10.1093/hmg/ddad052

[110] Tao L, Li D, Mu S, Tian G, Yan G. LncRNA MAPKAPK5_AS1 facilitates cell proliferation in hepatitis B virus -related hepatocellular carcinoma. Lab Invest. 2022;102(5):494–504. 10.1038/s41374-022-00731-9. 35264707 10.1038/s41374-022-00731-9

[111] Hu Y, Li Y, Xiong H, Zhang Y, Wang F, Zhuo W, Zeng Z, Zhao Y, Wang H, Hu P, Han S, Huang Y, Lv G, Zhao G. Exosomal SLC16A1-AS1-induced M2 macrophages polarization facilitates hepatocellular carcinoma progression. Int J Biol Sci. 2024;20(11):4341–63. 10.7150/ijbs.94440. 39247822 10.7150/ijbs.94440 PMC11379075

[112] He Z, Zhong Y, Regmi P, Lv T, Ma W, Wang J, Liu F, Yang S, Zhong Y, Zhou R, Jin Y, Cheng N, Shi Y, Hu H, Li F. Exosomal long non-coding RNA TRPM2-AS promotes angiogenesis in gallbladder cancer through interacting with PABPC1 to activate NOTCH1 signaling pathway. Mol Cancer. 2024;23(1):65. 10.1186/s12943-024-01979-z. 38532427 10.1186/s12943-024-01979-z PMC10967197

[113] Wang Z, He J, Bach DH, Huang YH, Li Z, Liu H, Lin P, Yang J. Induction of m(6)A methylation in adipocyte exosomal LncRNAs mediates myeloma drug resistance. J Exp Clin Cancer Res. 2022;41(1):4. 10.1186/s13046-021-02209-w. 34980213 10.1186/s13046-021-02209-w PMC8722039

[114] Chen J, Zhou Y, Wu M, Yuan Y, Wu W. m6A Modification Mediates Exosomal LINC00657 to Trigger Breast Cancer Progression Via Inducing Macrophage M2 Polarization. Clin Breast Cancer. 2023;23(5):546–60. 10.1016/j.clbc.2023.04.007. 37198028 10.1016/j.clbc.2023.04.007

[115] Xia X, Liu Y. Exosomes carrying lncRNA FLG-AS1 overexpression vectors inhibit the tumorigenesis of oral squamous cell carcinoma via fat mass and obesity-associated protein-mediated m6A modification. Cytotechnology. 2025;77(4):133. 10.1007/s10616-025-00801-y. 40611881 10.1007/s10616-025-00801-y PMC12214135

[116] Li T, Tian L, Cao J, Liu M. Cancer-associated fibroblasts secret extracellular vesicles to support cell proliferation and epithelial-mesenchymal transition in laryngeal squamous cell carcinoma. Mol Cell Probes. 2023;72:101934. 10.1016/j.mcp.2023.101934. 37777021 10.1016/j.mcp.2023.101934

[117] Yang Z, Luo Y, Zhang F, Ma L. Exosome-derived lncRNA A1BG-AS1 attenuates the progression of prostate cancer depending on ZC3H13-mediated m6A modification. Cell Div. 2024;19(1):5. 10.1186/s13008-024-00110-4. 38351022 10.1186/s13008-024-00110-4 PMC10863231

[118] Hu Z, Chen G, Zhao Y, Gao H, Li L, Yin Y, Jiang J, Wang L, Mang Y, Gao Y, Zhang S, Ran J, Li L. Exosome-derived circCCAR1 promotes CD8 + T-cell dysfunction and anti-PD1 resistance in hepatocellular carcinoma. Mol Cancer. 2023;22(1):55. 10.1186/s12943-023-01759-1. 36932387 10.1186/s12943-023-01759-1 PMC10024440

[119] Wei Y, Fu J, Zhang H, Ling Y, Tang X, Liu S, Yu M, Liu F, Zhuang G, Qian H, Zhang K, Yang P, Yang X, Yang Q, Ge S, Zhang B, Tan Y, Li L, Wang H. N6-methyladenosine modification promotes hepatocarcinogenesis through circ-CDYL-enriched and EpCAM-positive liver tumor-initiating exosomes. iScience. 2023;26(10):108022. 10.1016/j.isci.2023. 37954137 10.1016/j.isci.2023.108022 PMC10638478

[120] Liu X, Xiong H, Lu M, Liu B, Hu C, Liu P. Trans-3, 5, 4’-trimethoxystilbene restrains non-small-cell lung carcinoma progression via suppressing M2 polarization through inhibition of m6A modified circPACRGL-mediated Hippo signaling. Phytomedicine. 2024;126:155436. 10.1016/j.phymed.2024.155436. 38394728 10.1016/j.phymed.2024.155436

[121] Li P, Mi Q, Yan S, Xie Y, Cui Z, Zhang S, Wang Y, Gao H, Wang Y, Li J, Du L, Wang C. Characterization of circSCL38A1 as a novel oncogene in bladder cancer via targeting ILF3/TGF-β2 signaling axis. Cell Death Dis. 2023;14(1):59. 10.1038/s41419-023-05598-2. 36697384 10.1038/s41419-023-05598-2 PMC9876890

[122] Chen A, Zhang VX, Zhang Q, Sze KM, Tian L, Huang H, Wang X, Lee E, Lu J, Lyu X, Lee MJ, Wong CM, Ho DW, Ng IO. Targeting the oncogenic m6A demethylase FTO suppresses tumourigenesis and potentiates immune response in hepatocellular carcinoma. Gut. 2024;74(1):90–102. 10.1136/gutjnl-2024-331903. 38839271 10.1136/gutjnl-2024-331903 PMC11672076

[123] Ao K, Yin M, Lyu X, Xiao Y, Chen X, Zhong S, Wen X, Yuan J, Ye M, Zhang J, Li X, Hao Y, Guo X. METTL3-mediated HSPA9 m6A modification promotes malignant transformation and inhibits cellular senescence by regulating exosomal mortalin protein in cervical cancer. Cancer Lett. 2024;587:216658. 10.1016/j.canlet.2024.216658. 38253218 10.1016/j.canlet.2024.216658

[124] Zhang Y, Guo J, Zhang L, Li Y, Sheng K, Zhang Y, Liu L, Gong W, Guo K. CircASPH enhances exosomal STING to Facilitate M2 Macrophage Polarization in Colorectal Cancer. Inflamm Bowel Dis. 2023;29(12):1941–56. 10.1093/ibd/izad113. 37624989 10.1093/ibd/izad113

[125] Jin C, Gao J, Zhu J, Ao Y, Shi B, Li X. Exosomal NAT10 from esophageal squamous cell carcinoma cells modulates macrophage lipid metabolism and polarization through ac4C modification of FASN. Transl Oncol. 2024;45:101934. 10.1016/j.tranon.2024.101934. 38692194 10.1016/j.tranon.2024.101934 PMC11070927

[126] Gou J, Jia T, Song S, He J, Zhao D. M1 macrophage exosomes inhibit lung adenocarcinoma growth by up-regulating KLF6 via the reduction of ALKBH5-mediated KLF6 demethylation. Mol Cell Biochem. 2025. 10.1007/s11010-025-05432-7. 41348384 10.1007/s11010-025-05432-7

[127] Bridges MC, Daulagala AC, Kourtidis A. LNCcation: lncRNA localization and function. J Cell Biol. 2021' 10.1083/jcb.202009045 10.1083/jcb.202009045 PMC7816648 33464299

[128] Iaccarino I, Klapper W. LncRNA as cancer biomarkers. Methods Mol Biol. 2021;2348:27–41. 10.1007/978-1-0716-1581-2_2. 34160797 10.1007/978-1-0716-1581-2_2

[129] Gong H, Liu Z, Yuan C, Luo Y, Chen Y, Zhang J, Cui Y, Zeng B, Liu J, Li H, Deng Z. Identification of cuproptosis-related lncRNAs with the significance in prognosis and immunotherapy of oral squamous cell carcinoma. Comput Biol Med. 2024;171:108198. 10.1016/j.compbiomed.2024.108198. 38417385 10.1016/j.compbiomed.2024.108198

[130] Jin Y, Fan Z. New insights into the interaction between m6A modification and lncRNA in cancer drug resistance. Cell Prolif. 2024;57(4):e13578. 10.1111/cpr.13578. 37961996 10.1111/cpr.13578 PMC10984110

[131] Qin S, Mao Y, Chen X, Xiao J, Qin Y, Zhao L. The functional roles, cross-talk and clinical implications of m6A modification and circRNA in hepatocellular carcinoma. Int J Biol Sci. 2021;17(12):3059–79. 10.7150/ijbs.62767. 34421350 10.7150/ijbs.62767 PMC8375232

[132] Yang Y, Fan X, Mao M, Song X, Wu P, Zhang Y, Jin Y, Yang Y, Chen LL, Wang Y, Wong CC, Xiao X, Wang Z. Extensive translation of circular RNAs driven by N(6)-methyladenosine. Cell Res. 2017;27(5):626–41. 10.1038/cr.2017.31. 28281539 10.1038/cr.2017.31 PMC5520850

[133] Pinzani P, D’Argenio V, Del Re M, Pellegrini C, Cucchiara F, Salvianti F, Galbiati S. Updates on liquid biopsy: current trends and future perspectives for clinical application in solid tumors. Clin Chem Lab Med. 2021;59(7):1181–200. 10.1515/cclm-2020-1685. 33544478 10.1515/cclm-2020-1685

[134] Zhou B, Xu K, Zheng X, Chen T, Wang J, Song Y, Shao Y, Zheng S. Application of exosomes as liquid biopsy in clinical diagnosis. Signal Transduct Target Ther. 2020;5(1):144. 10.1038/s41392-020-00258-9. 32747657 10.1038/s41392-020-00258-9 PMC7400738

[135] Garcia-Romero N, Esteban-Rubio S, Rackov G, Carrión-Navarro J, Belda-Iniesta C, Ayuso-Sacido A. Extracellular vesicles compartment in liquid biopsies: clinical application. Mol Aspects Med. 2018;60:27–37. 10.1016/j.mam.2017.11.009. 29155161 10.1016/j.mam.2017.11.009

[136] Aheget H, Mazini L, Martin F, Belqat B, Marchal JA, Benabdellah K. Exosomes their role in pathogenesis, diagnosis and treatment of diseases. Cancers. 2020. 10.3390/cancers13010084 10.3390/cancers13010084 PMC7795854 33396739

[137] Bellassai N, D’Agata R, Jungbluth V, Spoto G. Surface plasmon resonance for biomarker detection: advances in non-invasive cancer diagnosis. Front Chem. 2019;7:570. 10.3389/fchem.2019.00570. 31448267 10.3389/fchem.2019.00570 PMC6695566

[138] Li B, Wu W, Xu W, Qian H, Ji C. Advances of extracellular vesicles isolation and detection frontier technology: from heterogeneity analysis to clinical application. J Nanobiotechnol. 2025;23(1):678. 10.1186/s12951-025-03768-2. 10.1186/s12951-025-03768-2 PMC12519844 41084034

[139] Xiao YL, Liu S, Ge R, Wu Y, He C, Chen M, Tang W. Transcriptome-wide profiling and quantification of N(6)-methyladenosine by enzyme-assisted adenosine deamination. Nat Biotechnol. 2023;41(7):993–1003. 10.1038/s41587-022-01587-6. 36593412 10.1038/s41587-022-01587-6 PMC10625715

[140] Körtel N, Rücklé C, Zhou Y, Busch A, Hoch-Kraft P, Sutandy FXR, Haase J, Pradhan M, Musheev M, Ostareck D, Ostareck-Lederer A, Dieterich C, Hüttelmaier S, Niehrs C, Rausch O, Dominissini D, König J, Zarnack K. Deep and accurate detection of m6A RNA modifications using miCLIP2 and m6Aboost machine learning. Nucleic Acids Res. 2021;49(16):e92. 10.1093/nar/gkab485. 34157120 10.1093/nar/gkab485 PMC8450095

[141] Pu Q, Ye Y, Hu J, Xie C, Zhou X, Yu H, Liao F, Jiang S, Jiang L, Xie G, Chen W. XNA probe and CRISPR/Cas12a-powered flexible fluorescent and electrochemical dual-mode biosensor for sensitive detection of m6A site-specific RNA modification. Talanta. 2023;252:123754. 10.1016/j.talanta.2022.123754. 36029686 10.1016/j.talanta.2022.123754

[142] Li Q, Sun C, Wang D, Lou J, RMNet. An RNA m6A cross-species methylation detection method for nanopore sequencing. Curr Drug Targets. 2025;26(11):799–812. 10.2174/0113894501405283250627072052. 40621754 10.2174/0113894501405283250627072052

[143] Kaplan JM, Wong HR. Biomarker discovery and development in pediatric critical care medicine. Pediatr Crit Care Med. 2011;12(2):165–73. 10.1097/PCC.0b013e3181. 20473243 10.1097/PCC.0b013e3181e28876 PMC2924462

[144] Rifai N, Gillette MA, Carr SA. Protein biomarker discovery and validation: the long and uncertain path to clinical utility. Nat Biotechnol. 2006;24(8):971–83. 10.1038/nbt1235. 16900146 10.1038/nbt1235

[145] Zhou J, Li L, Han Y, Ge G, Ji Q, Li H. RNA binding protein RALY facilitates colorectal cancer metastasis via enhancing exosome biogenesis in m6A dependent manner. Int J Biol Macromol. 2024;273(Pt 2):133112. 10.1016/j.ijbiomac.2024.133112. 38880454 10.1016/j.ijbiomac.2024.133112

[146] Nayak A, Khedri A, Chavarria A, Sanders KN, Ghalei H, Khoshnevis S. Sinefungin, a natural nucleoside analog of S-adenosyl methionine, impairs the pathogenicity of Candida albicans. NPJ Antimicrob Resist. 2024. 10.1038/s44259-024-00040-9 10.1038/s44259-024-00040-9 PMC11391927 39268078

[147] Yang WS, Kim JH, Jeong D, Hong YH, Park SH, Yang Y, Jang YJ, Kim JH, Cho JY. 3-Deazaadenosine, an S-adenosylhomocysteine hydrolase inhibitor, attenuates lipopolysaccharide-induced inflammatory responses via inhibition of AP-1 and NF-κB signaling. Biochem Pharmacol. 2020;182:114264. 10.1016/j.bcp.2020.114264. 33035507 10.1016/j.bcp.2020.114264

[148] Alshaer W, Zureigat H, Al Karaki A, Al-Kadash A, Gharaibeh L, Hatmal MM, Aljabali AAA, Awidi A, siRNA. Mechanism of action, challenges, and therapeutic approaches. Eur J Pharmacol. 2021;905:174178. 10.1016/j.ejphar.2021.174178. 34044011 10.1016/j.ejphar.2021.174178

[149] Zong Y, Lin Y, Wei T, Cheng Q. Lipid nanoparticle (LNP) enables mRNA delivery for cancer therapy. Adv Mater. 2023;35(51):e2303261. 10.1002/adma.202303261. 37196221 10.1002/adma.202303261

[150] Wolff RK, Blanchard JD, Preclinical Safety. J Aerosol Med Pulm Drug Deliv. 2025;38(3):136–44. 10.1089/jamp.2025.52511.isam. 40445826 10.1089/jamp.2025.52511.isam

[151] Shi T, Zhang H, Chen Y. The m6A revolution: transforming tumor immunity and enhancing immunotherapy outcomes. Cell Biosci. 2025;15(1):27. 10.1186/s13578-025-01368-z. 39987091 10.1186/s13578-025-01368-z PMC11846233

[152] Zhang XD, Sun J, Zheng XM, Zhang J, Tan LL, Fan LL, Luo YX, Hu YF, Xu SD, Zhou H, Zhang YF, Li H, Yuan Z, Wei T, Zhu HL, Xu DX, Xiong YW, Wang H. Plin4 exacerbates cadmium-decreased testosterone level via inducing ferroptosis in testicular Leydig cells. Redox Biol. 2024;76:103312. 10.1016/j.redox.2024.103312. 39173539 10.1016/j.redox.2024.103312 PMC11387904

[153] Meng X, Wang Y, Zhao W, Chen Y, Li W, Peng K, Xu H, Yang Y, Shan X, Huo W, Liu H, Ji F. Identification of differential m6A RNA methylomes and ALKBH5 as a potential prevention target in the developmental neurotoxicity induced by multiple sevoflurane exposures. Faseb j. 2024;38(14):e23793. 10.1096/fj.202400664R. 39003634 10.1096/fj.202400664R

[154] Ogawa A, Watanabe S, Ozerova I, Tsai AY, Kuchitsu Y, Chong HB, Kawakami T, Fuse J, Han W, Kudo R, Naito T, Sato K, Nakazawa T, Saheki Y, Hirayama A, Stadler PF, Arisawa M, Araki K, Bar-Peled L, Taguchi T, Sawa S, Inaba K, Wei FY. Adenosine kinase and ADAL coordinate detoxification of modified adenosines to safeguard metabolism. Cell. 2025;188(22):6151–69. 10.1016/j.cell.2025.07.041. 40840445 10.1016/j.cell.2025.07.041

[155] Moon H, Zang DY, Ryu MH, Seo Y, Oh B, Hwang S, Farrand L. FDA regulatory considerations for oncology drug development. Pharmacol Res Perspect. 2024;12(4):e1254. 10.1002/prp2.1254. 39075638 10.1002/prp2.1254 PMC11286537

[156] Zhao X, Hao R, Tong X, Zou L, Tang L, Zhang H, Xia L, Yang Z. Considerations on clinical development and regulatory of the oversea license-in anti-tumor drugs. Zhongguo Fei Ai Za Zhi. 2022;25(7):448–51. 10.3779/j.issn.1009-3419.2022.101.27 35899440 10.3779/j.issn.1009-3419.2022.101.27 PMC9346151

[157] Harvey BE. NASH: regulatory considerations for clinical drug development and U.S. FDA approval. Acta Pharmacol Sin. 2022;43(5):1210–4.10.1038/s41401-021-00832-z 10.1038/s41401-021-00832-z PMC9061714 35110697

[158] Zhang RR, Wang TT, Shen HY, Zhou X, Han QH, Li L, Zhang LY, Wang CA, Dong XT. Tumor microenvironment-responsive MnO x -mesoporous carbon nanoparticles for enhanced chemodynamic synergistic antitumor therapy. ACS Appl NANO Mater. 2025;8(6):2763–73. 10.1021/acsanm.4c06257.

