# Supplementary material for: The impact of m6A modified cargo conveyed by exosomes on the tumor microenvironment
Source: Discov Oncol. 2026 May 21;17:1215. doi: 10.1007/s12672-026-05060-7 (PMC13490356; doi:10.1007/s12672-026-05060-7)
Supplement: Supplementary file 1 [file 12672_2026_5060_MOESM1_ESM.docx]

**Risk of Bias assessment**

The results of the SYRCLE’s risk of bias assessment are presented in Table 4, and Tables 5 summarizing the analyses of the in vitro and in vivo sections, respectively. Although some of the questions were not answered in certain studies, these studies were not excluded from the systematic review. Their limitations have been highlighted and discussed in the Discussion section.

Table 4-Results of risk of bias assessment of in vitro section of included study.

| Article | 1 | 2 | 3 | 4 | 5 | 6 | 7 | 8 | 9 | 10 | Overall |
| --- | --- | --- | --- | --- | --- | --- | --- | --- | --- | --- | --- |
| Tong Liu et al. (2021)  (106) | U | Y | N/A | N/A | N/A | N/A | U | Y | Y | Y | Included |
| Chun Jin et al. (2024)  (125) | U | Y | N/A | N/A | U | N/A | U | Y | Y | Y | Included |
| Song Li et al. (2025)  (107) | U | Y | N/A | N/A | N/A | N/A | U | Y | Y | Y | Included |
| Bougras-Cartron et al.  (2023)  (108) | U | Y | N/A | N/A | N/A | N/A | U | U | U | U | Included |
| L. Xu et al. (2023)  (109) | U | N/A | N/A | N/A | N/A | N/A | N/A | Y | Y | Y | Included |
| Zhiqiang He et al. (2024)  (112) | U | Y | N/A | N/A | N/A | N/A | U | Y | Y | Y | Included |
| Lianyuan Tao et al. (2022)  (110) | N/A | N/A | N/A | N/A | N/A | N/A | N/A | Y | Y | Y | Included |
| Jiafeng Chen et al. (2023)  (114) | Y | U | N/A | N/A | U | N/A | U | Y | Y | Y | Included |
| Yuhang Hu et al. (2024)  (111) | U | U | N/A | N/A | U | N/A | U | Y | Y | Y | Included |
| Zongqiang Hu et al. (2023)  (118) | N/A | U | N/A | N/A | N/A | N/A | U | U | U | Y | Included |
| Yanping Wei et al. (2023)  (119) | U | Y | N/A | N/A | N/A | N/A | U | Y | Y | Y | Included |
| Ao Chen et al. (2024)  (122) | N/A | Y | N/A | N/A | U | N/A | U | Y | Y | Y | Included |
| Zhiming Wang et al. (2022)  (113) | N/A | Y | N/A | N/A | N/A | N/A | U | Y | Y | Y | Included |
| Zhi Yang et al. (2024)  (AIBG-AS1) | U | N | N/A | N/A | N/A | N/A | U | U | U | U | Included |
| Xiaoyang Xia & Yan Liu (2025)  (117) | U | Y | N/A | N/A | N/A | N/A | U | Y | Y | Y | Included |
| Xiaoyu Liu et al.  (2024)  (120) | N/A | Y | N/A | N/A | U | N/A | U | Y | Y | Y | Included |
| Peilong Li et al. (2023)  (121) | N/A | Y | N/A | N/A | U | N/A | U | Y | Y | Y | Included |
| Keyi Ao et al. (2024)  (123) | N/A | N/A | N/A | N/A | N/A | N/A | N/A | U | Y | Y | Included |
| Yuting Zhang, MD et al. (2023)  (124) | U | Y | N/A | N/A | U | N/A | U | Y | Y | Y | Included |
| Tingting Li et al. (2023)  (116) | U | Y | N/A | N/A | N/A | N/A | U | Y | Y | Y | Included |
| Jian Gou et al. (2023)  (126) | U | Y | N/A | N/A | N/A | N/A | N/A | U | Y | Y | Included |

N: No; N/A: Not applicable; U: Unclear; Y: Yes.

1. Was the allocation sequence adequately generated and applied?
2. Were the groups similar at baseline or were they adjusted for confounders in the analysis?
3. Was the allocation adequately concealed?
4. Were the animals randomly housed during the experiment?
5. Were the caregivers and/or investigators blinded from knowledge which intervention each animal received during the experiment?
6. Were animals selected at random for outcome assessment?
7. Was the outcome assessor blinded?
8. Were incomplete outcome data adequately addressed?
9. Are reports of the study free of selective outcome reporting?
10. Was the study apparently free of other problems that could result in high risk of bias?

Table 5-Results of risk of bias assessment of in vivo section of included study.

| Article | 1 | 2 | 3 | 4 | 5 | 6 | 7 | 8 | 9 | 10 | Overall |
| --- | --- | --- | --- | --- | --- | --- | --- | --- | --- | --- | --- |
| Tong Liu et al. (2021)  (106) | Y | Y | U | U | U | U | U | y | Y | Y | Included |
| Chun Jin et al. (2024)  (125) | Y | Y | U | U | U | U | U | Y | Y | Y | Included |
| Song Li et al. (2025)  (107) | U | Y | U | U | U | U | U | Y | Y | Y | Included |
| L. Xu et al. (2023)  (109) | U | U | U | U | U | U | U | Y | Y | U | Included |
| Lianyuan Tao et al. (2022)  (110) | U | U | U | U | U | U | U | Y | Y | Y | Included |
| Zhiqiang He et al. (2024)  (112) | U | Y | U | U | U | U | U | Y | Y | Y | Included |
| Zhiming Wang et al. (2022)  (113) | U | Y | U | U | U | U | U | Y | Y | Y | Included |
| Yuhang Hu et al. (2024)  (111) | Y | Y | U | U | U | U | U | Y | Y | Y | Included |
| Zongqiang Hu et al. (2023)  (118) | U | U | U | U | U | U | U | Y | U | Y | Included |
| Yanping Wei et al. (2023)  (119) | U | Y | U | U | U | U | U | Y | Y | Y | Included |
| Ao Chen et al. (2024)  (122) | U | Y | U | U | U | U | U | Y | Y | Y | Included |
| Xiaoyu Liu et al.  (2024)  (120) | U | U | U | U | U | U | U | Y | Y | Y | Included |
| Peilong Li et al. (2023)  (121) | Y | Y | U | U | U | U | U | Y | Y | Y | Included |
| Keyi Ao et al. (2024)  (123) | U | Y | U | U | U | U | U | Y | Y | U | Included |
| Yuting Zhang, MD et al. (2023)  (124) | U | Y | U | U | U | U | U | Y | Y | Y | Included |
| Tingting Li et al. (2023)  (116) | U | Y | U | U | U | U | U | U | Y | Y | Included |
| Jian Gou et al. (2023)  (126) | U | U | U | U | U | U | U | Y | Y | U | Included |

N: No; N/A: Not applicable; U: Unclear; Y: Yes.

1. Was the allocation sequence adequately generated and applied?
2. Were the groups similar at baseline or were they adjusted for confounders in the analysis?
3. Was the allocation adequately concealed?
4. Were the animals randomly housed during the experiment?
5. Were the caregivers and/or investigators blinded from knowledge which intervention each animal received during the experiment?
6. Were animals selected at random for outcome assessment?
7. Was the outcome assessor blinded?
8. Were incomplete outcome data adequately addressed?
9. Are reports of the study free of selective outcome reporting?
10. Was the study apparently free of other problems that could result in high risk of bias?
